# TGF-β downregulation-induced cancer cell death is finely regulated by the SAPK signaling cascade

**DOI:** 10.1038/s12276-018-0189-8

**Published:** 2018-12-06

**Authors:** Zhezhu Han, Dongxu Kang, Yeonsoo Joo, Jihyun Lee, Geun-Hyeok Oh, Soojin Choi, Suwan Ko, Suyeon Je, Hye Jin Choi, Jae J. Song

**Affiliations:** 1 Institute for Cancer Research, Yonsei University College of Medicine, Seoul, Korea; 2 Department of Oncology, Affiliated Hospital of Yanbian University, Yanji, Jilin Province P.R. China; 3 Severance Biomedical Science Institute, Yonsei University College of Medicine, Seoul, Korea; 4 Department of Internal Medicine, Yonsei University College of Medicine, Seoul, Korea

## Abstract

Transforming growth factor (TGF)-β signaling is increasingly recognized as a key driver in cancer. In progressive cancer tissues, TGF-β promotes tumor formation, and its increased expression often correlates with cancer malignancy. In this study, we utilized adenoviruses expressing short hairpin RNAs against TGF-β1 and TGF-β2 to investigate the role of TGF-β downregulation in cancer cell death. We found that the downregulation of TGF-β increased the phosphorylation of several SAPKs, such as p38 and JNK. Moreover, reactive oxygen species (ROS) production was also increased by TGF-β downregulation, which triggered Akt inactivation and NOX4 increase-derived ROS in a cancer cell-type-specific manner. We also revealed the possibility of substantial gene fluctuation in response to TGF-β downregulation related to SAPKs. The expression levels of Trx and GSTM1, which encode inhibitory proteins that bind to ASK1, were reduced, likely a result of the altered translocation of Smad complex proteins rather than from ROS production. Instead, both ROS and ROS-mediated ER stress were responsible for the decrease in interactions between ASK1 and Trx or GSTM1. Through these pathways, ASK1 was activated and induced cytotoxic tumor cell death via p38/JNK activation and (or) induction of ER stress.

## Cancer: Tipping the signaling balance

Reducing the levels of the multifunctional protein transforming growth factor (TGF)-β in cancer cells prevents tumor growth in mice. Previous studies have shown that high levels of TGF-β in cancerous tissue are associated with accelerated disease progression. Hye Jin Choi and Jae J Song at Yonsei University in Seoul, South Korea, and colleagues infected cancer cells with genetically modified viruses that reduced the expression of the gene encoding TGF-β. The resulting decrease in TGF-β protein led to cell death by stimulating the production of reactive oxygen species and signaling through the apoptosis signal-regulating kinase 1 (ASK1) pathway. When tumor-bearing mice were infected with these modified viruses, their overall survival was improved. Further understanding the mechanisms through which TGF-β regulates cancer cell survival will contribute to the development of new approaches in cancer treatment.

## Introduction

The transforming growth factor (TGF) superfamily comprises three isoforms of multifunctional cytokines (namely, β1, β2, and β3) that regulate numerous cellular and biological functions, including cell proliferation, apoptosis, differentiation, and migration; embryonic patterning; stem cell maintenance; immune regulation; bone formation; and tissue remodeling and repair^1–3^. The wide variety of TGF-β functions is highly cell-type specific and context dependent^1,4^. For example, TGF-β acts as a tumor suppressor in normal and early cancer cells by promoting apoptosis over proliferation, thus hindering immortalization^5^. On the other hand, it also promotes tumor metastasis by stimulating the epithelial–mesenchymal transition, chemoattraction, migration, invasion, and cell adhesion^6–10^. The mechanisms by which TGF-β inhibits cell proliferation while promoting cell growth and enhancing both stem cell pluripotency and differentiation remain an enigma^11–13^.

TGF-β binds to two types of serine/threonine kinase receptors^14^, type I and type II, which form heteromeric cell surface complexes that stimulate the canonical (Smad-dependent) signaling pathway^10^. Activation of type I receptors leads to C-terminal phosphorylation of Smad2 and Smad3, which then dissociate and form a heterotrimeric complex with Smad4^15,16^. This complex then translocates to the nucleus to regulate target gene expression^17,18^. TGF-β can also stimulate Smad-independent signaling pathways, which involve the activation of small GTP-binding protein Rho^19^, phosphatidylinositol 3-kinase (PI3K)-Akt^20–22^, and TGF-β-activated kinase 1 (TAK1)^23^, as well as Ras-extracellular signal–regulated kinase (ERK), c-Jun N-terminal kinase (JNK), and p38 stress-activated protein kinase (SAPK)^24–26^.

JNK and p38 are also activated by apoptosis signal-regulating kinase 1 (ASK1), a mitogen-activated protein kinase (MAPK) kinase kinase^27,28^. However, the roles of JNK and p38 signaling pathways during apoptosis have been controversial depending on the duration or strength of the signals^29,30^. The activation of ASK1 is mainly triggered under cytotoxic stresses by the tumor necrosis factor Fas and reactive oxygen species (ROS)^28,31–33^.

ROS are formed as a natural by-product of oxygen metabolism^34^. Large amounts of ROS are produced via multiple mechanisms, depending on the cell and tissue type^35^. Elevated levels of ROS have been detected in almost all cancers, in which they promote many aspects of tumor development and progression^36^. However, ROS can induce cancer cell apoptosis as well as senescence^36^. Additionally, low doses of hydrogen peroxide and superoxide have been shown to stimulate cell proliferation in a wide variety of cancer cell types^37^. Recently, it was shown that ROS can trigger endoplasmic reticulum (ER) stress or vice versa in vivo and in vitro^38,39^. Under prolonged and severe ER stress, the unfolded protein response (UPR) can become cytotoxic. Among the UPR signaling pathways, inositol-requiring enzyme 1α (IRE1α) and protein kinase RNA-like kinase (PERK) are predominantly represented as sensors of ER stress^40,41^. Likewise, oxidative stress-sensing redox proteins such as thioredoxin (Trx) play a role in many important biological processes, including redox signaling^42^. Trx has antiapoptotic effects, including a direct inhibitory interaction with ASK1^43^. The redox state-dependent association and dissociation of Trx with ASK1 lead to MAPK activation-induced apoptosis^44^. The activity of ASK1 is also suppressed by glutathione *S*-transferase Mu 1 (GSTM1), an enzyme involved in the metabolism of drugs and xenobiotics^45^. Similar to Trx, GSTM1 protects cells from a variety of stresses, including oxidative stress^45^.

In this study, we designed adenoviruses to deliver short hairpin RNAs (shRNAs) for TGF-β1 or TGF-β2 and found that downregulation of TGF-β1 or TGF-β2 leads to tumor cell death by inducing ASK1 activation and p38 and JNK phosphorylation through an ER stress/ROS-escalated positive feedback circuit.

## Materials and methods

### Cell culture

Human A549 (lung adenocarcinoma), U251N (glioblastoma), DU-145 (prostate cancer), Huh-7 (hepatocarcinoma), MDA-MB-231 (breast carcinoma), MDA-MB-231-Her2 (Her2-stably transfected breast carcinoma), SK-Hep1 (hepatocarcinoma), A375 (melanoma), HPAC (pancreas adenocarcinoma), MiaPaCa-2 (pancreas adenocarcinoma), and human embryonic kidney 293A cells were cultured in Dulbecco’s modified Eagle’s medium (DMEM, HyClone, Logan, UT, USA) containing 10% fetal bovine serum (FBS, HyClone, Logan, UT, USA). Human normal pancreatic cells (Applied Biological Materials, Canada) were cultured in Prigrow II medium containing 10% FBS. Cells were maintained in a 37 °C humidified atmosphere containing 5% CO_2_.

### Reagents

Small interfering RNAs (siRNAs) for NOX4, NOX1, Smad4, and ASK1 and antibodies against glyceraldehyde 3-phosphate dehydrogenase, phospho-HSP27 (Ser78), HSP27, Trx, GSTM1, AP1, SP1, Smad4, ASK1, and phospho-ASK1 (Thr845) were purchased from Santa Cruz Biotechnology (Santa Cruz, CA, USA). Antibodies against p38, phospho-p38 (Thr180/Tyr182), phospho-ERK (Thr202/Tyr204), phospho-src (Tyr416), phospho-Akt (Ser473), phospho-stat3 (Tyr705), phospho-p65 (Ser536), phospho-JNK (Thr183/Tyr185), phospho-Smad2 (Ser465/467), phospho-Smad3 (Ser423/425), Smad2, Smad3, p65, ERK, Akt, stat3, JNK, src, BIP, Calnexin, Ero1-Lα, CHOP, PERK, and PDI were purchased from Cell Signaling Technology (Beverly, MA, USA). Phospho-IRE1α was purchased from Thermo Fisher Scientific (Waltham, MA, USA). Peroxiredoxin (Prx) was purchased from Abfrontier (Seoul, Korea). Glutaredoxin (Grx) was purchased from Novus Biologicals (Littleton, CO, USA). Recombinant human TGF-β1 and TGF-β2 were purchased from R&D Systems (Minneapolis, MN, USA). SB203580 was purchased from EMD Millipore (Billerica, MA, USA), and GKT137831 was purchased from ApexBio (Houston, TX, USA). All other chemicals were purchased from Sigma-Aldrich (St. Louis, MO, USA).

### Construction of shuttle vectors expressing human TGF-β1 and TGF-β2 shRNA

**Fig. 1:**
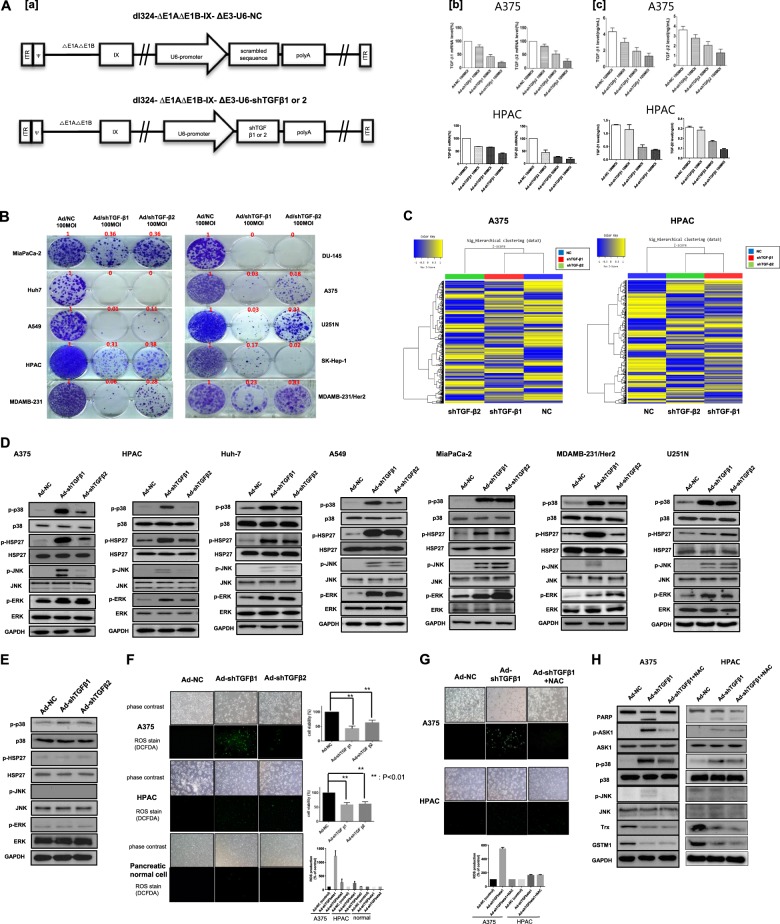
TGF-β downregulation and ROS generation by TGF-β shRNAs. **A** Downregulation of TGF-β1 and TGF-β2 after infection with adenoviruses expressing TGF-β shRNAs. **a** Schematic structure of adenoviral vectors expressing TGF-β shRNA. dl324-∆E1A-∆E1B-∆E3-IX-U6-NC (Ad-NC) is a replication-incompetent adenovirus used as the negative control. It contains the scrambled DNA sequence for shRNA, which is under the control of the U6 promoter. dl324-∆E1A-∆E1B∆E3-IX-U6-shTGF-β1 or -β2 (Ad-shTGF-β1 or -β2) is a replication-incompetent adenovirus expressing human TGF-β1 or -β2 shRNA. NC negative control. **b** Downregulation of human transforming growth factor β1 (TGF-β1) or β2 by adenovirus expressing shTGF-β1 or -β2. Human A375 and HPAC cells were infected with various MOIs of adenovirus expressing shRNA targeting human TGF-β1 (Ad-shTGF-β1) or human TGF-β2 (Ad-shTGF-β2) or scrambled RNA (Ad-NC). TGF-β1 or -β2 mRNA was assayed by quantitative real-time polymerase chain reaction (qRT-PCR), and **c** protein levels were assayed by enzyme-linked immunosorbent assay (ELISA). MOI multiplicity of infection, NC negative control. Error bars represent the standard error from three independent experiments. **B** Various cancer cell lines were treated with adenovirus expressing shTGF-β1 or -β2 for 48 h and incubated for an additional 14 days for clonogenic assays. The numbers indicate the relative ratio of clone numbers to those of Ad-NC. **C** Global transcriptome analysis of TGF-β downregulation. Expression values of differentially expressed genes are shown in heat map format. Gene expression levels are visualized as row standardized *z* scores ranging from green (−1) to red (+1) across all samples. The rows are organized by hierarchical clustering analysis with complete linkage and Euclidean distance as a measure of similarity from samples of A375 cells (NC, shTGF-β1, shTGF-β2) (Left) and HPAC cells (NC, shTGF-β1, shTGF-β2) (Right). NC, A375, and HPAC cells infected with adenovirus expressing nonsense shRNA as a negative control at 100 MOI; shTGF-β1 or -β2; A375 and HPAC cells infected with adenoviruexpressing TGF-β1 or -β2 shRNA at 100 MOI. **D** Effect of adenovirus expressing shTGF-β1 or shTGF-β2 in various cancer cell lines. Each cell line was treated with adenovirus expressing shTGF-β1 or shTGF-β2 at 100 MOI. After 48 h, the expression levels of p-p38, p38, p-HSP27, HSP27, p-ERK, ERK, p-JNK, JNK, and GAPDH were detected via western blot analysis. **E** Pancreatic normal cell lines were treated with adenovirus expressing shTGF-β1 or -β2 at 100 MOI. After 48 h, the expression levels of p-p38, p38, p-HSP27, HSP27, p-ERK, ERK, p-JNK, JNK, and GAPDH were detected via western blot analysis. **F** ROS generation was induced by shTGF-β1- and shTGF-β2-expressing adenoviruses. A375 cells, HPAC cancer cells, and pancreatic normal cells were infected with adenovirus expressing shTGF-β1 or -β2 at 100 MOI, respectively, after 48 h of incubation with DCF-DA (20 μM, 1 h) for the detection of ROS using a fluorescent reader and microscopy. Cell viability was tested via an MTS viability assay after 48 h of infection. Error bars represent the standard error from three independent experiments. *p* Values <0.01 indicate a very significantly different viability of Ad-shTGF-β1 or -β2 compared to the control (Ad-NC). **G** Effects of NAC treatment with adenovirus expressing TGF-β1 or -β2 shRNA in melanoma and pancreatic cancer cells. A375 (top) or HPAC (bottom) cells were infected with adenovirus expressing shTGF-β1 or -β2 at 100 MOI, respectively. After 6 h, infected cells were treated with NAC (10 mM) for 42 h and then incubated with DCF-DA (20 μM, 1 h) for the detection of ROS using a fluorescent reader and microscopy. **H** A375 and HPAC cells were infected with adenovirus expressing shTGF-β1 or -β2. After 6 h, infected cells were treated with NAC (10 mM) for 42 h, and then the expression levels of PARP, p-ASK1, ASK1, p-p38, p38, p-JNK, JNK, Trx, and GAPDH were detected by western blot analysis

The detailed construction of human TGF-β1 and TGF-β2 shRNA was described in Oh et al.^46^. The target sequence of TGF-β1 (5’-ACCAGAAATACAGCAACAATTCCTG-3’) was selected after validation among three candidate sequences (Suppl. Figure 1A). The target sequence of TGF-β2 (5’-GGATTGAGCTATATCAGATTCTCAA-3’) was selected after validation among five candidate sequences (Supple. Figure 1B). To express human TGF-β1 shRNA in adenovirus, the top strand sequence (5’-GATCCGCCAGAAATACAGCAACAATTCCTGTCTCTCCAGGAATTGTTGCTGTATTTCTGGTTTTTTTA-3’) and the bottom strand sequence (5’-AGCTTAAAAAAACCAGAAA TACAGCAACAATTCCTGGAGAGACAGGAATTGTTGCTGTATTTCTGGTG-3’) were annealed and subcloned into BamHI/HindIII-digested pSP72ΔE3-U6 E3 shuttle vector to generate pSP72ΔE3-U6-shTGF-β1. To express human TGF-β2 shRNA in adenovirus, the top strand sequence (5’-GATCCGGATTGAGCTATATCAGATTCTCAATCTCTTGAGAATCTGATATAGCTCAATCCTTTTA-3’) and the bottom strand sequence (5’- AGCTTAAAAGGATTGAGCTATATCAGATTCTCAAGAGATTGAGAATCTGATATAGCTCAATCCG-3’) were annealed and subcloned into BamHI/HindIII-digested pSP72ΔE3-U6 E3 shuttle vector to generate pSP72ΔE3-U6-shTGF-β2.

### Production of adenoviral vectors

These vectors, named pSP72ΔE3-U6-shTGF-β1 or pSP72ΔE3-U6-shTGF-β2, were linearized by XmnI digestion and co-transformed into *Escherichia coli* BJ5183 together with the SpeI-digested adenoviral vector (dl324-IX) for homologous recombination. The recombined adenoviral plasmids dl324-IX-ΔE3-U6-NC, dl324-IX-ΔE3-U6-shTGF-β1, and dl324-IX-ΔE3-U6-shTGF-β2 were then digested with PacI and transfected into 293A cells to generate replication-incompetent adenovirus (Ad-NC, Ad-shTGF-β1, and Ad-shTGF-β2).

### Names of the recombinant adenoviruses

Ad-NC, negative control adenovirus

Ad-shTGF-β1, adenovirus expressing shRNA for human TGF-β1

Ad-shTGF-β2, adenovirus expressing shRNA for human TGF-β2

### MTS viability assay

The CellTiter 96® Aqueous Assay Kit (Promega, Madison, WI, USA) is composed of solutions of a novel tetrazolium compound (3-(4,5-dimethylthiazol-2-yl)-5-(3-carboxymethoxyphenyl)-2-(4-sulfophenyl)-2H-tetrazolium, inner salt (MTS)) and an electron coupling reagent (phenazine ethosulfate). MTS is bioreduced by cells into a formazan product that is soluble in tissue culture media. After adenovirus (NC, shT1, shT2) infection at a multiplicity of infection (MOI) of 100 for 48 h to A375 or HPAC cell lines in 96-well plates, a total of 50 μL of supernatant from each well was transferred into a new 96-well flat-bottom plate. The absorbance of the formazan at 490 nm was measured directly from 96-well assay plates without additional processing. The conversion of MTS into aqueous, soluble formazan is accomplished by dehydrogenase enzymes found in metabolically active cells. The quantity of formazan product as measured by the absorbance at 490 nm is directly proportional to the number of living cells in culture.

### Western blot analysis

Cells were lysed with 1× Laemmli lysis buffer (62.5 mM Tris, pH 6.8, 2% sodium dodecyl sulfate, 10% glycerol, 0.002% bromophenol blue), and the protein concentration was determined using a BCA Protein Assay Kit (Thermo Scientific, Fremont, CA, USA). Then protein samples were separated by sodium dodecyl sulfate-polyacrylamide gel electrophoresis (SDS-PAGE), and the gels were electro-transferred onto a polyvinylidene difluoride membrane (Millipore, Billerica, MA, USA). Each membrane was blocked with 5% nonfat dry milk in phosphate-buffered saline (PBS)-Tween-20 (0.1%, v/v) at room temperature for 1 h. The membrane was then incubated with primary antibody (diluted according to the manufacturer’s instructions) for 2 h. Horseradish peroxidase-conjugated anti-rabbit or anti-mouse immunoglobulin G (IgG) was used as a secondary antibody. Immunoreactive proteins were visualized with a chemiluminescent detection kit (ELPIS biotech, Daejon, Korea). For imaging, a ChemiDoc system (Syngene, Frederick, MD, USA) was used.

### Real-time polymerase chain reaction (RT-PCR)

After infection of adenovirus expressing shRNA for TGF-β1 or -β2 at an MOI of 100, cells were lysed with Trizol reagent (Life Technologies, Carlsbad, CA, USA), and the total RNA was isolated using chloroform. The RNA concentration was determined using a Nanodrop 2000 (Thermo Scientific). The real-time PCR was performed using a Power SYBR Green RNA-to-CT 1-Step Kit (Life Technologies). The reaction mixture contained the reverse transcriptase enzyme mix, reverse transcription PCR mix, forward primer, reverse primer, RNA template, and nuclease-free water. Human TGF-β1 cDNA was amplified using the forward primer 5’-TTGCTTCAGCTCCACAGAGA-3’ and the reverse primer 5’-TGGTTGTAGAGGGCAAGGAC-3’. Human TGF-β2 cDNA was amplified using the forward primer 5’-GTGAATGGCTCTCCTTCGAC-3’ and the reverse primer 5’-CCTCGAGCTCTTCGCTTTTA-3’. Human NOX4 cDNA was amplified using the forward primer 5’- AGGAGAACCAGGAGATTGTTGGATAAA-3’ and the reverse primer 5’- ATCTGAGGGATGACTTATGACCGAAAT-3’. Human β-actin was amplified using the forward primer 5’-GGCTGTATTCCCCTCCATCG-3’ and the reverse primer 5’-CCAGTTGGTAACAATGCCATGT-3’.

### Microarray analyses

cDNA microarray was performed using total RNA isolated from two different cancer cell lines after 48 h of infection with TGF-β1 or -β2 shRNA-expressing adenovirus. RNA was prepared using Trizol (Invitrogen LifeTechnologies). All cDNA microarray experiments, including cRNA labeling, hybridization to the Illumina expression bead-chip, scanning, and data analyses using Illumina GenomeStudiov2011.1, were performed by Macrogen Inc. (Seoul, Korea).

### Clonogenic assay

For survival determination, cells were plated on 6-well plates at 1 × 10^5^ cells/well. After adenovirus (NC, shT1, shT2) infection at an MOI of 100 for 48 h in A375 or HPAC cell lines, cells were trypsinized and replated in the wells of 6-well plates at 5 × 10^3^ or 1 × 10^4^ cells/well. Then the cells were monitored daily by microscopy. When cells formed colonies, the remaining cells on the plate were fixed with 4% paraformaldehyde and stained with 0.5% crystal violet.

### Measurement of intracellular levels of ROS

Intracellular ROS were assessed using the ROS-specific probe 2’-7’-diclorofluorescein diacetate (DCF-DA, Sigma-Aldrich, St. Louis, MO, USA). Cells were incubated with 20 μM DCF-DA for 1 h, and fluorescence signals were obtained with a fluorescence microscope (Olympus IX71).

### Enzyme-linked immunosorbent assay (ELISA)

Cells were plated in the wells of 6-well plates at 1 × 10^5^ cells/well. After 48 h, the supernatants were collected. The levels of secreted TGF-β1 or TGF-β2 were determined by ELISA according to the manufacturer’s instructions (R&D Systems, Minneapolis, MN, USA).

### Site-directed mutagenesis

HA-tagged ASK1 wild type was constructed in the pCDNA3.1 plasmid (Invitrogen, Carlsbad, CA). A kinase-inactive form of ASK1 (K709M) was constructed from PCR using pcDNA3.1-HA-ASK1 as a template and the QuickChange II Site-Directed Mutagenesis Kit (Agilent Technologies, Santa Clara, SF, USA). The sense and antisense primers used were 5′-GCAACCAAGTCAGAATTGCTATTATGGAAATCCCAGAGAGAGAC-3′ and 5′-GTCTCTCTCTGGGATTTCCATAATAGCAATTCTGACTTGGTTGC-3′, respectively, for K709M.

### Immunoprecipitation (IP)

Co-IP procedures were performed at 4 °C unless otherwise indicated, using a Pierce spin column that can be capped and plugged with a bottom plug for incubation or unplugged to remove the supernatant by centrifugation at 1000 × *g* for 1 min. Binding of Trx, GSTM1, ASK1, AP1, SP1, or Smad4 antibody to protein A/G agarose was performed using the protocol described in Pierce crosslink IP kits with slight modification. Protein A/G agarose slurry (20 µl) was washed twice with 200 µl of PBS buffer and incubated with 100 µl of Trx, GSTM1, ASK1, AP1, SP1, or Smad4 antibody prepared in PBS (10 µl of Trx, GSTM1, ASK1, AP1, SP1, or Smad4 antibody + 85 µl of H_2_O + 5 µl of 20× PBS) at 25 °C for 30 min on a mixer. In parallel, mouse or rabbit serum with the same concentration of IgG was similarly prepared as a negative control (NC). Then the supernatant was discarded, and the beads were washed three times with 300 µl of PBS, followed by incubation with 50 µl of disuccinimidyl suberate (DSS) solution (2.5 µl of 20× PBS + 38.5 µl of H_2_O + 2.5 mM DSS in dimethyl sulfoxide) at 25 °C for 45–60 min on a mixer. After removing the supernatant, the beads were washed three times with 50 µl of 100 mM glycine (pH 2.8), twice with 300 µl of PBS buffer containing 1% NP-40 and then once with 300 µl of PBS. The antibody-crosslinked beads were incubated overnight at 4 °C with 600 µl of lysate of A375 or HPAC cells that was pre-cleared with control agarose resin (Pierce) for 1 h on a shaker. After removing the supernatant (flow-through) and washing with 300 µl of washing buffer (25 mM Tris, 150 mM NaCl, 1 mM EDTA, 1% NP-40, 5% glycerol, pH 7.4) three times, the immunoprecipitates were eluted with 60 µl of elution buffer and then boiled for 10 min. The eluted complex was subjected to SDS-PAGE separation for western blotting.

### Chromatin IP assay (ChIP assay)

The ChIP assay was performed using a kit from Thermo Scientific (Thermo Fisher Scientific, Waltham, USA) according to the manufacturer’s instructions. Briefly, following treatment, cells were washed with PBS, crosslinked with 1% formaldehyde for 10 min, rinsed with ice-cold PBS, collected into PBS containing protease inhibitors, and then resuspended in lysis buffer (1% SDS, 10 mM EDTA, 50 mM Tris at pH 8.1 with 1% protease inhibitor cocktail). After the cells were sonicated to produce 200–1000-bp DNA fragments, followed by centrifugation to remove insoluble material, IP was performed with the indicated antibodies overnight at 4 °C with mixing. Then 20 μL of protein A/G beads was added to each IP and incubated for 2 h at 4 °C with mixing. After removing the supernatant, the beads were washed twice with IP Wash Buffer 1, once with Wash Buffer 2, once with 150 μL of 1× IP Elution Buffer, and then incubated at 65 °C for 30 min with vigorous shaking. After dispensing the supernatant (containing the eluted protein–chromatin complex) into prepared tubes with NaCl and Proteinase K in a 65 °C heat block for 1.5 h, 750 μL of DNA Binding Buffer was added, and 500 μL of each sample was removed into a DNA Clean-Up Column, which was inserted into a 2 mL collection tube. After removing the supernatant (flow-through), the remaining sample was dispensed into the same DNA Clean-Up Column and washed once with Wash Buffer, and 50 μL of DNA Column Elution Solution was added to the column. After centrifugation, the resulting solution was the purified DNA. RT-PCR was performed using primers specific for the human Trx promoter (5’-TCCAGGAGTCTGCCTCTGTTAG-3’ and 5’-CTGCTGGA GTCTGACGAGCG-3’) and the GSTM1 promoter (5’-TAGGATCTGGCTGGTGTCTC-3’ and 5’-GTGCGGATTCCGCAGACAGG-3’). PCR using Absolute qPCR SYBR Green Fluorescein Mix (Thermo Scientific) was performed as follows: 40 cycles of 95 °C for 15 s and 62 °C for 1 min, with an initial incubation at 95 °C for 15 min.

### Confocal immunofluorescence staining

Cells were fixed with 4% formaldehyde for 15 min and then washed three times with PBS. After washing with PBS 3 times, the slides were incubated with 5% bovine serum albumin (BSA) for 1 h. After blocking, the slides were incubated in primary antibody (Smad4) at the appropriate dilution (1:100) in PBS for 12 h at 4 °C and then washed three times with PBS. After washing with PBS 3 times, the slides were incubated in Flamma 552-conjugated secondary antibody (1:1000) (BioActs, Korea) in PBS for 1 h at room temperature and then washed three times with PBS. After washing with PBS 3 times, the slides were counterstained with DAPI-Fluoromount-G (Aviva, San Diego, USA) for 20 min at room temperature to stain nuclei and then coverslipped. Images were acquired using a confocal laser scanning microscope (Carl Zeiss, Jena, Germany).

### Nucleus/cytosol fractionation

Cells were lysed with 0.5% Triton X-100 lysis buffer (50 mM Tris-HCI (pH 7.5), 0.5% Triton X-100, 137.5 mM NaCI, 10% glycerol, 1 mM sodium vanadate, 5 mM EDTA, and protease inhibitors (1 mM phenylmethanesulfonylfluoride)) on ice for 15 min and then centrifuged at 3000 rpm for 5 min. After centrifugation, the supernatant (membrane/cytoplasmic fraction) was transferred into a new microcentrifuge tube, and the nuclear pellets were rinsed with lysis buffer and centrifuged at 13,000 rpm for 15 min. After centrifugation, the supernatants were transferred into a new microcentrifuge tube, and then an equal amount of 2× SDS-PAGE sample buffer was added to the tubes containing the nuclear and membrane/cytoplasmic fractions. Both tubes were boiled for 10 min, and each boiled sample was subjected to SDS-PAGE separation for western blotting.

### Animal study

To generate a xenograft tumor model, 8 × 10^6^ A375 or HPAC cells were injected into the subcutaneous abdominal region of male BALB/c athymic nude mice. When the tumors reached an average size of 60–80 mm^3^, the nude mice received intratumoral injections of 1 × 10^9^ plaque-forming units (pfu) of oncolytic adenovirus diluted in 50 μL of PBS or PBS alone. The adenoviruses used were defective control adenovirus (Ad-shNC) and TGF-β1 or TGF-β2 shRNA-expressing defective adenoviruses (Ad-sh TGF-β1, Ad-shTGF-β2). Intratumoral injection was repeated three times every other day. Regression of tumor growth was assessed by taking measurements of the length (*L*) and width (*W*) of the tumor. Tumor volume was calculated using the following formula: volume = 0.52 × *L* × *W*^2^.

### Immunohistochemistry (IHC)

For IHC, tumor tissues were extracted, fixed for 24 h in 10% formaldehyde, and embedded in paraffin. IHC staining was performed as follows. Tissue sections were deparaffinized twice with xylene for 10 min and were rehydrated using a graded alcohol series. After removing endogenous peroxidases using 0.1% H_2_O_2_, sections were washed three times with PBS. Antigen retrieval was achieved by incubating the sections in 10 mM citrate buffer (pH 6.0) (DAKO, Glostrup, Denmark) using a microwave oven. Then the sections were permeabilized with 0.5% PBX (0.5% Triton X-100 in PBS) for 30 min and washed three times with PBS. After blocking for 1 h with 5% BSA, the primary antibody was added, and the sections were incubated overnight at 4 °C. Primary Antibody Enhancer (Thermo Fisher Scientific, Waltham, MA, USA) and horseradish peroxidase Polymer (Thermo Scientific) were used for signal amplification. To develop the colored product, a mixture of DAB (3,3′-diaminobenzidine) Plus Chromogen and DAB Plus Substrate (Thermo Fisher Scientific) was added for 5 min. After washing with PBS, 20% hematoxylin counterstain was added for 2–5 min to stain the nuclei. Finally, the tissue sections were dehydrated in a graded alcohol series. After clearing twice in xylene, the tissue sections were coverslipped with mounting media (xylene:mount = 1:1) for microscopy.

### TUNEL assay

To measure in situ apoptosis, a terminal deoxynucleotidyl transferase-mediated dUTP nick end labeling (TUNEL) assay was performed using tumor tissue sections prepared as described for IHC. The TUNEL assay was carried out according to the manufacturer’s instructions (Promega, Madison, WI, USA).

### Statistical analysis

The data are expressed as the mean ± standard error (SE). Differences between groups were examined using unpaired two-tailed *t* tests. Statistical comparison was made using the Graph Pad (Systat Software Inc.). *p* Values <0.05 were considered statistically significant (*p < 0.05; **p < 0.01).

## Results

### Downregulation of TGF-β1 and TGF-β2 after infection with adenoviruses expressing shRNAs of TGF-β

Almost all human tumors overexpress TGF-β, primarily TGF-β1 and TGF-β2, which contribute to the induction of tumor cell invasion and metastasis^47^. To decrease the expression of TGF-β1 and TGF-β2, we infected human melanoma (A375) or pancreatic cancer (HPAC) cells with recombinant adenoviruses containing shRNA for TGF-β1 or TGF-β2 (shTGF-β1 or shTGF-β2, respectively) at various MOIs (10, 50, and 100). The shRNA sequences used in the experiment after screening several candidate target sequences originated from Oh et al.^46^ (Suppl. Figure 1A, B). The mRNA and protein levels of TGF-β1 and TGF-β2 were measured by real-time PCR and ELISA, respectively. The results show that TGF-β1 mRNA levels were suppressed by 80% at an MOI of 100, at which TGF-β2 mRNA levels were suppressed by 75% (Fig. 1a, b). Similarly, the protein levels of TGF-β1 and TGF-β2 were also reduced (Fig. 1a, c).

### shTGF-β1 and shTGF-β2 induce the phosphorylation of SAPKs

**Fig. 2:**
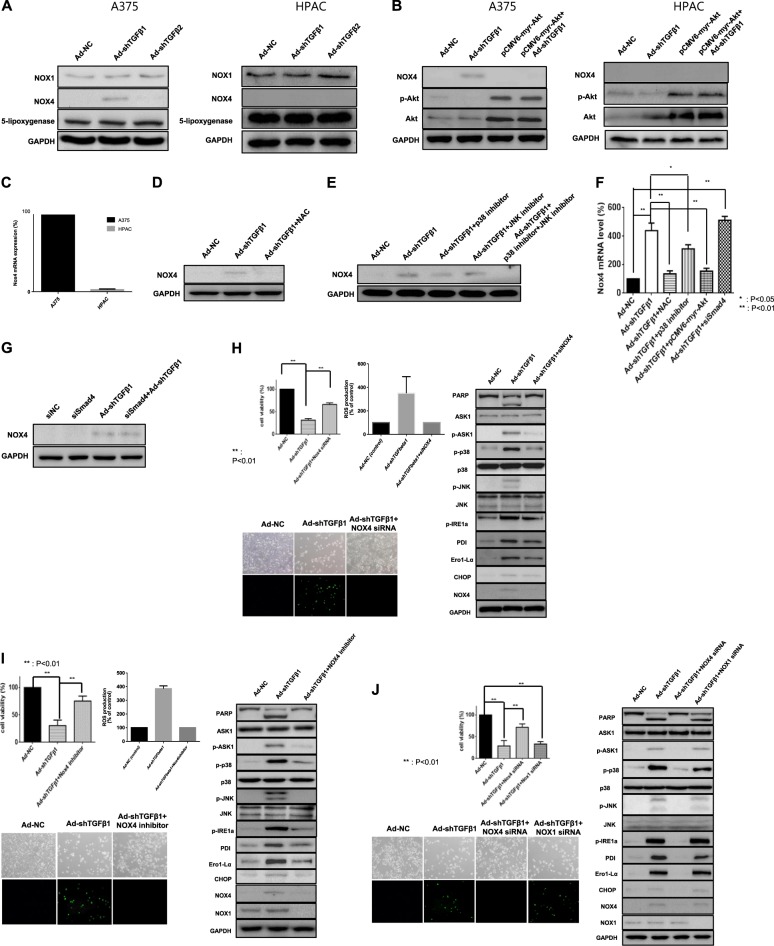
NOX4 is responsible for TGF-β downregulation-induced ROS generation. **a** A375 and HPAC cells were infected with adenovirus expressing shTGF-β1 or -β2 at 100 MOI, respectively. After 48 h, the expression levels of NOX1, NOX4, 5-lipoxygenase, and GAPDH were detected by western blot analysis. **b** A375 and HPAC cells were infected with adenovirus expressing shTGF-β1 or transfected with pCMV6-myr-Akt or infected with adenovirus expressing shTGF-β1 and subsequently transfected with pCMV6-myr-Akt. After 48 h, NOX4, phospho-Akt, Akt, and GAPDH were detected by western blot analysis. **c** Expression levels of NOX4 mRNA in A375 and HPAC cells were assayed by quantitative real-time polymerase chain reaction (qRT-PCR). **d** A375 cells were infected with adenovirus expressing shTGF-β1. After 6 h, infected cells were treated with NAC (10 mM) for 42 h, and then the expression of NOX4 and GAPDH was detected by western blot analysis. **e** A375 cells were infected with adenovirus expressing shTGF-β1 at 100 MOI, and after 6 h, infected cells were treated with p38 inhibitor (SB203580, 10 μM) or JNK inhibitor (SP600125, 10 μM) or both for 42 h. Then the expression of NOX4 and GAPDH was detected by western blot analysis. **f** A375 cells were infected with adenovirus expressing shTGF-β1 at 100 MOI or adenovirus expressing shTGF-β1 followed by NAC (10 mM) or adenovirus expressing shTGF-β1 followed by p38 inhibitor (SB203580, 10 μM) or adenovirus expressing shTGF-β1 followed by transfection with pCMV6-myr-Akt or adenovirus expressing shTGF-β1 followed by transfection with siRNA of Smad4 based on the siRNA transfection protocol (Santa Cruz, CA, USA). After 48 h, the expression levels of NOX4 mRNA were assayed by quantitative real-time polymerase chain reaction (qRT-PCR). Error bars represent the standard error from three independent experiments. Asterisks indicate a significant difference compared to each given control (*p < 0.05; **p < 0.01). **g** A375 cells were infected with adenovirus expressing shTGF-β1 at 100 MOI or adenovirus expressing shTGF-β1 followed by transfection with siRNA of Smad4. After 48 h, NOX4 and GAPDH were detected by western blot analysis. **h** A375 cells were infected with adenovirus expressing shTGF-β1 at 100 MOI or adenovirus expressing shTGF-β1 followed by transfection with siRNA of NOX4 based on the siRNA transfection protocol (Santa Cruz, CA, USA). After 48 h, cell viability was tested via an MTS viability assay (upper left), or cells were incubated with DCF-DA (20 μM, 1 h) for the detection of ROS using a fluorescent reader and microscopy (upper right, lower left) or the expression levels of various ER stress markers; in addition, PARP, p-ASK1, ASK1, p-p38, p38, p-JNK, and JNK were detected by western blot analysis (right). **i** A375 cells were infected with adenovirus expressing shTGF-β1 at 100 MOI or adenovirus expressing shTGF-β1 followed by treatment with NOX4 inhibitor (GKT137831, 140 nM). After 48 h, cell viability was tested via an MTS viability assay (upper left), or cells were incubated with DCF-DA (20 μM, 1 h) for the detection of ROS using a fluorescent reader and microscopy (upper right, lower left) or the expression levels of various ER stress markers; in addition, PARP, p-ASK1, ASK1, p-p38, p38, p-JNK, and JNK were detected by western blot analysis (right). **j** A375 cells were infected with adenovirus expressing shTGF-β1 at 100 MOI or adenovirus expressing shTGF-β1 followed by transfection with siRNA of NOX4 or NOX1 based on the siRNA transfection protocol (Santa Cruz, CA, USA). After 48 h, cell viability was tested via an MTS viability assay (upper left), or cells were incubated with DCF-DA (20 μM, 1 h) for the detection of ROS using a fluorescent reader and microscopy (upper right, lower left) or the expression levels of various ER stress markers; in addition, PARP, p-ASK1, ASK1, p-p38, p38, p-JNK, and JNK were detected by western blot analysis (right)

Because high expression of TGF-β is correlated with malignancy in many cancers^3^, we investigated the effect of TGF-β downregulation in cancer cell lines. We first examined whether shTGF-β1 or shTGF-β2 could inhibit cancer cell survival using a clonogenic assay, which can measure long-term cancer cell survival. The results of the clonogenic assay revealed that the survival of various cancer cells was reduced after infection with adenoviruses carrying shTGF-β1 or shTGF-β2 (Fig. 1b). To examine the impact of TGF-β downregulation on the overall gene fluctuation, a global transcriptomic response was assessed by microarray. Figure 1c shows clearly different patterns of *z* scores after clustering analysis between control cells (A375 and HPAC cells) and TGF-β-downregulated control cells, suggesting a possibility of substantial gene fluctuation toward cell death after TGF-β downregulation (Supple. Figure 2). To further demonstrate that cell death by TGF-β downregulation is also caused by SAPK pathways, we examined various key signaling molecules of SAPK pathways. We observed that the levels of phosphorylated-p38 (phospho-p38), phosphorylated-HSP27 (phospho-HSP27), phosphorylated-JNK (phospho-JNK), and phosphorylated-ERK (phospho-ERK) were increased by shTGF-β1 and shTGF-β2 (Fig. 1d). However, after TGF-β downregulation in normal pancreatic cells, the phosphorylation levels of various key signaling pathway molecules, including HSP27, JNK, and ERK, did not change (Fig. 1e). To determine whether shTGF-β1 or shTGF-β2 produced off-target effects, we analyzed A375 cells infected with adenoviruses carrying the shRNAs and supplemented the cells with recombinant TGF-β protein. Western blot analyses showed that the addition of recombinant TGF-β1 protein restored the levels of phospho-src, phospho-p65, phospho-stat3, phospho-HSP27, and phospho-p38 (Suppl. Figure 3A). Additionally, morphological recovery was also observed (Suppl. Figure 3B). Similar patterns were also observed with recombinant TGF-β2 protein (Suppl. Figure 3C, D), suggesting that there were no off-target effects of shTGF-β1 or shTGF-β2. Then we investigated whether TGF-β itself could also activate p38 in the cancer cells in which TGF-β downregulation activated p38. Supplementary figure 4 shows the weak activation of p38 in cancer cells and specific survival molecules, for example, p65 in A375 cells or Akt and stat3 in HPAC cells, after TGF-β1 treatment with no complete sign of cell death.

### shTGF-β1 and shTGF-β2 induce ROS generation in cancer cells

ROS are known to be related to JNK and p38 pathway activation^48–50^. Thus we assessed the generation of ROS in A375 and HPAC cells infected with adenoviruses carrying shRNAs for TGF-β1 and TGF-β2. We found that knockdown of TGF-β1 greatly stimulated the generation of ROS in A375 cells 48 h after adenovirus infection. In contrast, few ROS were generated in A375 cells after knockdown of TGF-β2 or in HPAC cells after knockdown of TGF-β1 or TGF-β2, and ROS were barely detectable in normal pancreatic cells after TGF-β downregulation (Fig. 1f), which was primarily derived from low infection efficiency in normal pancreatic cells (data not shown). *N-*acetylcysteine (NAC) is an aminothiol and a synthetic precursor of intracellular cysteine and glutathione and is thus considered a strong antioxidant^51^. Because shTGF-β1 induced ROS in A375 cancer cells, we next evaluated the effect of NAC on cell growth and apoptosis. We found that, as predicted, NAC treatment reduced ROS levels in A375 cells. (Fig. 1g). Furthermore, cell death was inhibited (Fig. 1g, h), suggesting that ROS production was responsible for the cancer cell death induced by TGF-β1 downregulation; however, cell death still occurred without much ROS generation in HPAC cells (Fig. 1g, h).

### NOX4 is responsible for ROS generation induced by the downregulation of TGF-β

Next, we examined various ROS-generating enzymes, including NOX1, NOX4, and 5-lipoxygenase. Nicotinamide adenine dinucleotide phosphate oxidase is one of the main sources implicated in the production of cellular ROS^52,53^. Among the ROS-generating enzymes, NOX4 was strongly expressed after TGF-β1 downregulation but not after TGF-β2 downregulation (Fig. 2a), and its expression induced by TGF-β1 downregulation was almost completely repressed by constitutive Akt activation in A375 cells (Fig. 2b). However, NOX4 was not detected in HPAC cells. Figure 2c shows that NOX4 mRNA levels in HPAC cells, compared with those in A375 cells, were barely detectable. Intriguingly, NOX4 expression was also repressed by ROS inhibition or p38 inhibition, which are downstream molecules of NOX4 (Fig. 2d–f), suggesting that a positive feedback loop was formed to sustain cancer cell death in A375 cells. However, the Smad signaling pathway was not involved in NOX4 generation (Fig. 2f, g). To demonstrate NOX4 function as an indispensable part of TGF-β1 downregulation-induced cancer cell death in A375 cells, both NOX4 siRNA and GKT137831 (ApexBio, Houston, TX, USA) as a NOX4 inhibitor were used to determine whether its inhibition could reverse TGF-β1 downregulation-induced ROS generation/ER stress and subsequent cancer cell death. Figure 2h, i demonstrated that NOX4 was an essential mediator of TGF-β1 downregulation-induced cancer cell death in A375 cells. However, owing to the nature of dual NOX1/NOX4 inhibition of GKT137831, NOX1 siRNA was also used to check the possibility of NOX1 involvement in TGF-β1 downregulation-induced ROS generation/ER stress and subsequent cancer cell death. As a result, NOX1 was not found to be involved in this pathway (Fig. 2j).

### ASK1 functions as a key mediator of TGF-β1 downregulation-induced cell death

**Fig. 3:**
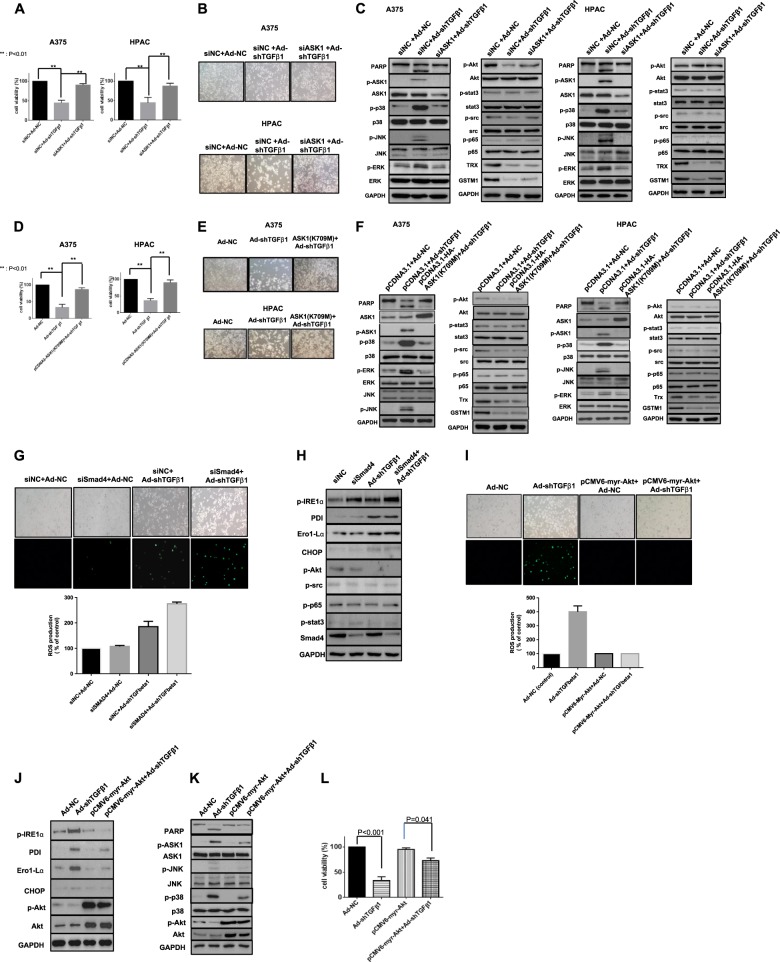
ASK1 functions as a key mediator of TGF-β downregulation-induced cell death triggered by non-canonical signaling Akt inactivation **a** ASK1 mediates TGF-β-induced cell death via p38/JNK activation. A375 and HPAC cells were infected with adenovirus expressing shTGF-β1 or -β2 (100 MOI) and subsequently transfected with siASK1. siASK1 transfection was based on the siRNA transfection protocol (Santa Cruz, CA, USA). After 48 h, cell viability was tested by an MTS viability assay. Error bars represent the standard error from three independent experiments. Asterisks indicate a significant difference compared to each given control (*p < 0.05; **p < 0.01). **b** A375 and HPAC cells were infected with adenovirus expressing shTGF-β1 or -β2 and subsequently transfected with siASK1. After 48 h, morphological changes were observed using microscopy. **c** A375 and HPAC cells were infected with adenovirus expressing shTGF-β1 or -β2 and subsequently transfected with siASK1. After 48 h, the expression levels of p-p38, p-Akt, p-HSP27, HSP27, p-ERK, p-src, p-p65, p-JNK, p-stat3, and GAPDH were detected by western blot analysis. **d** A375 and HPAC cells were infected with adenovirus expressing shTGF-β1 or -β2 and subsequently transfected with ASK1 kinase mutant ASK1 (K709M). After 48 h, cell viability was tested with an MTS viability assay. Error bars represent the standard error from three independent experiments. Asterisks indicate a significant difference compared to each given control (*p < 0.05; **p < 0.01). **e** A375 and HPAC cells were infected with adenovirus expressing shTGF-β1 or -β2 and subsequently transfected with ASK1 kinase mutant ASK1 (K709M). After 48 h, morphological changes were observed using microscopy. **f** A375 and HPAC cells were infected with adenovirus expressing shTGF-β1 or -β2 and subsequently transfected with ASK1 kinase mutant ASK1 (K709M). After 48 h, the expression levels of p-p38, p-Akt, p-HSP27, HSP27, p-ERK, p-src, p-p65, p-JNK, p-stat3, and GAPDH were detected by western blot analysis. **g** A375 cells were infected with adenovirus expressing shTGF-β1 and subsequently transfected with siSmad4. After 48 h of incubation with DCF-DA (20 μM, 1 h), ROS were detected using a fluorescent reader and microscopy. **h** After 48 h, the expression of various ER stress markers in addition to survival-related molecules was detected by western blot analysis; A375 cells were infected with adenovirus expressing shTGF-β1 and subsequently transfected with pCMV6-myr-Akt. **i** After 48 h of incubation with DCF-DA (20 μM, 1 h), ROS were detected using a fluorescent reader and microscopy. **j** After 48 h, the expression levels of various ER stress markers and phospho-Akt and Akt were detected by western blot analysis. **k** After 48 h, the expression levels of PARP, p-ASK1, ASK1, p-p38, p38, p-JNK, JNK, and GAPDH were detected by western blot analysis. **l** After 48 h, cell viability was tested via an MTS viability assay. Error bars represent the standard error from three independent experiments. Decreased *p* value to 0.041 from <0.001 after Akt activation followed by TGF-β1 downregulation means a significant decrease in cell death

As ASK1 and SAPKs were activated during TGF-β1 downregulation-induced apoptosis in A375 and HPAC cells, we investigated whether the ASK1 signaling cascade was the main regulator of this pathway. As shown in Fig. 3, downregulation of ASK1 using ASK1 siRNA restored the viability (Fig. 3a) and morphology (Fig. 3b) of cells following TGF-β downregulation. Furthermore, ASK1 siRNA also reversed SAPK activation, including ASK1 activation. Interestingly, survival signals including Akt, src, or stat3 activation and Trx/GSTM1 showed nearly no recovery (Fig. 3c). To confirm that ASK1 activation drives cell death following TGF-β downregulation, we constructed and utilized an inactive ASK1 mutant, ASK1 (K709M). Similar results were also obtained using this mutant and ASK1 inhibition using siRNA (Fig. 3d–f).

### Non-canonical signaling through Akt inactivation by TGF-β downregulation triggers ROS–ASK1 axis activation

Then we investigated the source of ROS–ASK1 axis activation and found that the Smad signaling pathway was not involved in TGF-β downregulation-induced ROS generation (Fig. 3g) or ER stress (Fig. 3h). Therefore, PI3K-Akt, as a well-known non-canonical TGF-β signaling pathway, was investigated to determine whether it was the main source of ROS generation using a constitutively active form of Akt, N-terminally myristoylation signal-attached Akt (myr-Akt). As expected, TGF-β downregulation-induced ROS generation was greatly decreased by Akt activation (Fig. 3i). The decrease in ROS caused by Akt activation also decreased both ER stress and ASK1–p38/JNK cascade-induced apoptosis (Fig. 3j–l). These results support the strong correlation between the lack of effect on Akt inactivation and the loss of NOX4 expression/trivial ROS production following TGF-β1 downregulation in HPAC cells

### TGF-β downregulation decreases Trx and GSTM1 expression and the formation of complexes with ASK1

**Fig. 4:**
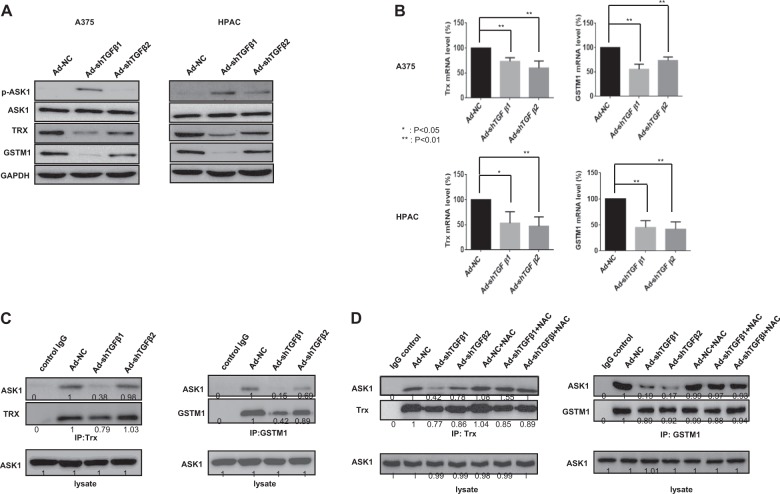
Decreases in Trx and GSTM1 expression and the formation of complexes with ASK1 after TGF-β downregulation. **a** A375 and HPAC cells were infected with adenovirus expressing shTGF-β1 or -β2 at 100 MOI, respectively. After 48 h, the expression levels of p-ASK1, ASK1, Trx, GSTM1 and GAPDH were detected by western blot analysis. **b** A375 and HPAC cells were infected with adenovirus expressing shTGF-β1 or -β2 at 100 MOI, respectively. After 48 h, the expression levels of Trx and GSTM1 mRNA were assayed by quantitative real-time polymerase chain reaction (qRT-PCR). Error bars represent the standard error from three independent experiments. Asterisks indicate a significant difference compared to each given control (*p < 0.05; **p < 0.01). **c** A375 cells were infected with adenovirus expressing shTGF-β1 or -β2 at 100 MOI. After 48 h, lysates were then subjected to immunoprecipitation with an anti‐Trx (Left) or anti‐GSTM1 (Right) antibody to identify changes in complex formation. The numbers indicate the relative band intensity to that of Ad-NC. **d** A375 cells were infected with adenovirus expressing shTGF-β1 or -β2 at 100 MOI. After 6 h, infected cells were treated with NAC (10 mM) for 42 h. Then lysates were subjected to immunoprecipitation with an anti‐Trx (Left) or anti‐GSTM1 (Right) antibody to identify changes in complex formation. The numbers indicate the relative band intensity to that of Ad-NC after measuring band intensity using a densitometer

Next, we investigated how HPAC cells die concomitant with ASK1 activation and trivial ROS production. We found that TGF-β downregulation decreased both the mRNA and protein levels of Trx and GSTM1, both of which act as negative ASK1-interacting regulators by inhibiting ASK1 kinase activity and cytotoxicity in both A375 and HPAC cells (Fig. 4a, b). Moreover, the interactions between Trx and ASK1 and between GSTM1 and ASK1 were also reduced by TGF-β downregulation, particularly by TGF-β1 downregulation (Fig. 4c). In contrast, NAC treatment restored the interactions between Trx and ASK1 and between GSTM1 and ASK1, which is likely related to the repression of ROS production (Fig. 4d). These results strongly suggest that an increase in ASK1 activity follows both reduced Trx and GSTM1 expression and the dissociation of ASK1–Trx and ASK1–GSTM1 complexes. However, this increased ASK1 activity leads to HPAC cancer cell death only by reduced Trx and GSTM1 expression.

### Regulation of Trx and GSTM1 promoter activity and AP-1, Sp1, and Smad expression by TGF-β

**Fig. 5:**
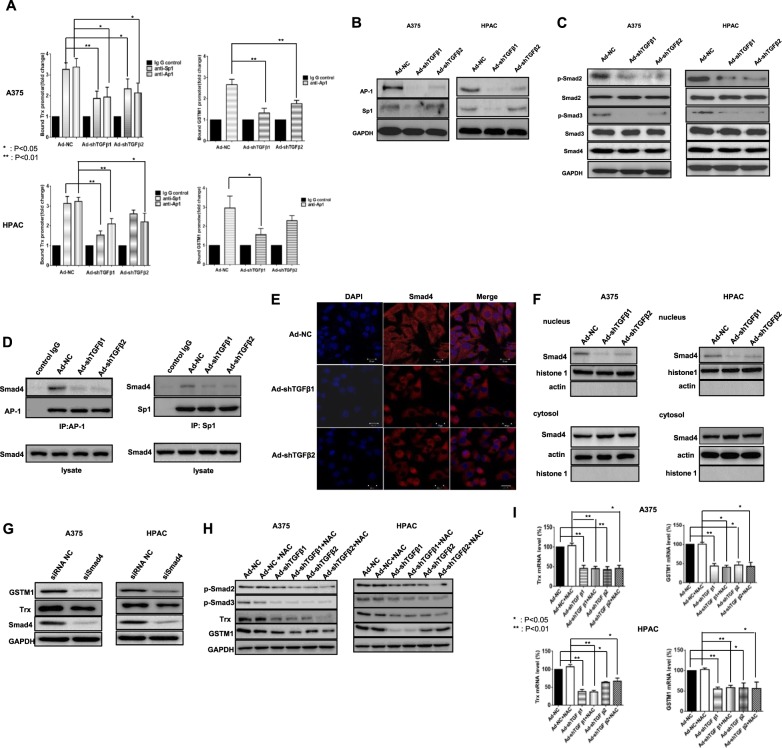
Regulation of Trx and GSTM1 promoter activity by TGF-β downregulation via a Smad complex. **a** A375 and HPAC cell lines were infected with adenovirus expressing shTGF-β1 or -β2 at 100 MOI, respectively. After 48 h, Trx and GSTM1 promoter activities were analyzed by Chip assays using antibodies to AP-1 or Sp1. Error bars represent the standard error from three independent experiments. Asterisks indicate a significant difference compared to each given control (*p < 0.05; **p < 0.01). **b** A375 and HPAC cells were infected with adenovirus expressing shTGF-β1 or -β2 at 100 MOI, respectively. After 48 h, the expression levels of AP-1, Sp1 and GAPDH were detected by western blot analysis using whole-cell lysates. **c** p-Smad2, Smad2, p-Smad3, Smad3, Smad4, and GAPDH were detected by western blot analysis. **d** A375 cells were infected with adenovirus expressing shTGF-β1 or -β2 at 100 MOI. After 48 h, lysates were then subjected to immunoprecipitation with an anti‐AP1 or anti-Sp1 antibody to identify changes in Smad4 in complex formation. **e** A375 cells were infected with adenovirus expressing shTGF-β1 or -β2 at 100 MOI. After 48 h, Smad4 localization was examined by confocal immunofluorescence staining. Representative confocal immunofluorescence staining of each infected A375 was performed as described in the Materials and methods section to confirm the localization of Smad4. **f** A375 and HPAC cells were infected with adenovirus expressing shTGF-β1 or -β2 at 100 MOI, respectively. After 48 h, Smad4 localization was examined in nuclear or cytosolic fractionation using nuclear (histone 1) or cytosol (actin) markers. **g** A375 and HPAC cell lines were transfected with negative control siRNA or Smad4 siRNA. After 48 h, transfected cells were detected with GSTM1, Trx, Smad4, and GAPDH. **h** Effects of NAC on Trx and GSTM1 expression in melanoma and pancreatic cancer cells expressing shTGF-β1 or -β2 adenovirus. A375 and HPAC cells were infected with adenovirus expressing shTGF-β1 or -β2 at 100 MOI, respectively. After 6 h, infected cells were treated with NAC (10 mM) for 42 h. Then, p-Smad2, p-Smad3, Trx, GSTM1, and GAPDH were detected by western blot analysis. **i** A375 and HPAC cells were infected with adenovirus expressing shTGF-β1 or -β2 at 100 MOI, respectively. After 6 h, infected cells were treated with NAC (10 mM) for 42 h. Then the expression levels of Trx and GSTM1 mRNA were assayed by quantitative real-time polymerase chain reaction (qRT-PCR). Error bars represent the standard error from three independent experiments. Asterisks indicate a significant difference compared to each given control (*p < 0.05; **p < 0.01)

Because TGF-β downregulation suppressed Trx and GSTM1 expression, we examined their promoter sequences to identify the possible mechanism underlying this finding. The Trx promoter contains several consensus AP-1 (21) and Sp1 (2) binding sites, whereas the GSTM1 promoter contains AP-1-binding sites (30) but not the Sp1-binding site, as revealed by the Champion ChiP transcription factor search program supplied by Qiagen (Germantown, MD, USA). We found that TGF-β downregulation decreased Trx and GSTM1 promoter binding by Sp1 and Ap1 proteins (Fig. 5a), most likely due to decreased AP-1 and Sp1 protein levels (Fig. 5b). As canonical TGF-β signaling primarily regulates gene transcription via Smads^54^, we investigated the levels of activated Smad proteins following infection with adenoviruses expressing shRNAs for TGF-β1 or TGF-β2. Western blot analysis showed that the expression levels of phospho-Smad2 and phospho-Smad3 were decreased (Fig. 5c). In addition, IP results also demonstrated reduced interactions of AP-1 and Sp1 with Smad proteins (Fig. 5d), suggesting that the reduction in the Smad complex, including Smad4, makes it difficult to mobilize to the nucleus, where these proteins act as transcription factors. In support of the inability of Smad4 to translocate to the nucleus during TGF-β downregulation, Smad4 localization in the nucleus was reduced, as observed by confocal immunofluorescence staining (Fig. 5e), and the reduced amount of Smad4 in the nucleus during TGF-β downregulation was further confirmed by nucleus/cytosol fractionation (Fig. 5f). Smad4 downregulation confirmed that Smad4 is a key mediator of TGF-β downregulation-induced Trx and GSTM1 suppression (Fig. 5g). However, intriguingly, NAC treatment did not recover the original state of phosphorylated Smad2, Smad3, or GSTM1/Trx prior to TGF-β downregulation (Fig. 5h, i). These data suggest that the reduced expression of Trx and GSTM1 results from decreased levels of their transcriptional binding proteins, such as AP-1 and Sp1 and their subsequent reduced interactions with Smad proteins rather than from ROS generation.

### phospho-JNK/phospho-p38 functions as a bridge from ROS to the ASK1 axis in a positive feedback activation loop

**Fig. 6:**
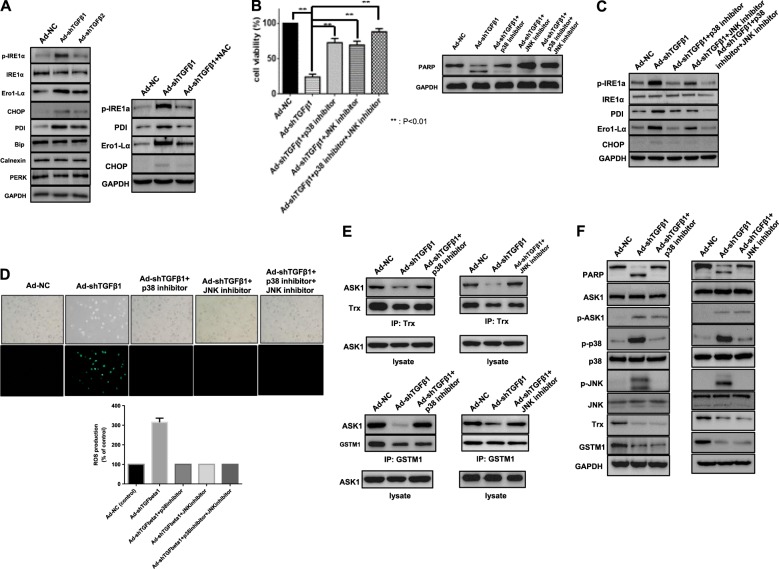
Involvement of reciprocal ER stress and SAPK activation during TGF-β downregulation-induced cell death. **a** A375 cells were infected with adenovirus expressing shTGF-β1 or -β2. After 48 h, various ER stress-related proteins were detected by western blot analysis. **b** A375 cells were infected with adenovirus expressing shTGF-β1 at 100 MOI, and after 6 h, infected cells were treated with a JNK inhibitor (SP600125, 10 μM) or a p38 inhibitor (SB203580, 10 μM) for 42 h. Then cell viability was tested using an MTS viability assay (left). Error bars represent the standard error from three independent experiments. Asterisks indicate a significant difference compared to each given control (**p < 0.01). Additionally, the expression levels of PARP and GAPDH were detected by western blot analysis (right). **c** A375 cells were infected with adenovirus expressing shTGF-β1 at 100 MOI, and after 6 h, infected cells were treated with a JNK inhibitor (SP600125, 10 μM) or a p38 inhibitor (SB203580, 10 μM) for 42 h. Then ER stress-related proteins that responded to TGF-β downregulation (Fig. 5a) were detected by western blot analysis. **d** A375 cells were infected with adenovirus expressing shTGF-β1 at 100 MOI, and after 6 h, infected cells were treated with a JNK inhibitor (SP600125, 10 μM) or a p38 inhibitor (SB203580, 10 μM) for 42 h and then incubated with DCF-DA (20 μM, 1 h) for the detection of ROS using a fluorescent reader and microscopy. **e** A375 cell lines were infected with adenovirus expressing shTGF-β1 at 100 MOI, and after 6 h, infected cells were treated with a JNK inhibitor (SP600125, 10 μM) or a p38 inhibitor (SB203580, 10 μM) for 42 h. Then lysates were subjected to immunoprecipitation using an anti-Trx or anti-GSTM1 antibody to identify changes in ASK1. **f** A375 cells were infected with adenovirus expressing shTGF-β1 at 100 MOI, and after 6 h, infected cells were treated with a JNK inhibitor (SP600125, 10 μM) or a p38 inhibitor (SB203580, 10 μM) for 42 h. Then the expression levels of PARP, ASK1, p-ASK1, p-p38, p38, p-JNK, JNK, Trx, GSTM1, and GAPDH were detected by western blot analysis

To investigate the possibility of ROS-mediated ER stress in TGF-β downregulation, various ER stress-related molecules were examined. Higher expression levels of ER stress markers (CHOP, PDI, and Ero1-Lα) and increased phosphorylation of ER stress regulators (IRE1α and phospho-PERK) were observed (Fig. 6a, left). The increase in ER markers was reduced by repression of ROS by NAC (Fig. 6a, right), and p38/JNK inhibition significantly recovered cell viability (Fig. 6b) with a concurrent decrease in ER stress-related molecules and ROS production (Fig. 6c, d). Moreover, similar events also occurred in cells after p38/JNK inhibition: a decrease in ASK1-Trx/GSTM1 dissociation (Fig. 6e) along with a decrease in PARP cleavage (Fig. 6f). Taken together, these results suggest that p-p38/p-JNK and ROS cooperatively enhanced TGF-β downregulation-induced cell death through persistent ER stress in A375 cells with NOX4 expression.

### Adenovirus expressing TGF-β shRNA increases tumor regression in a mouse model

**Fig. 7:**
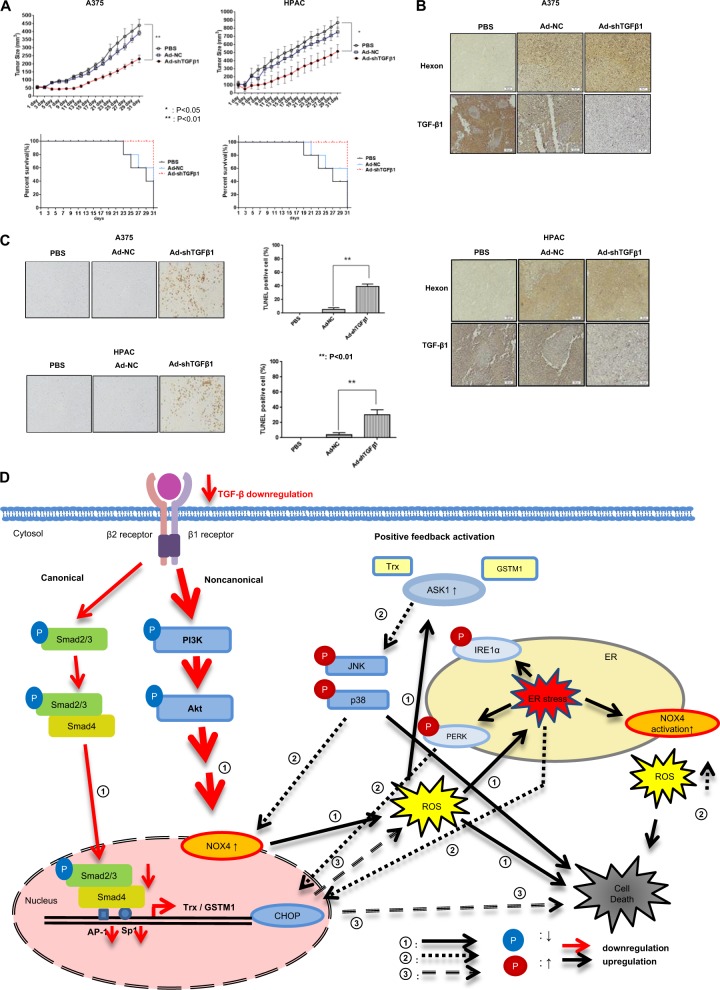
Antitumor effect and schematic diagram of cancer cell death by TGF-β downregulation. **a** BALB/c athymic nude mice were injected with 8 × 10^6^ A375 or HPAC cells in 100 μL. When the tumors reached an average size of 60–80 mm^3^, the nude mice received intratumoral injections of 1 × 10^9^ plaque-forming units (pfu) of various kinds of adenovirus (Ad-NC, Ad-shTGF-β1, or Ad-shTGF-β2) in 50 μL of PBS or PBS alone on days 1, 3, and 5. Tumor volume was monitored and recorded every 2 days until the end of the study. Values represent the mean ± SE (five animals per group) (top). Asterisks indicate a significant difference compared to each given control (*p < 0.05; **p < 0.01). Overall survival was determined throughout a 31-day time course (bottom). **b** Representative immunohistochemical analysis of recombinant adenovirus-infected tumor sections was performed as follows. Three animals per group of BALB/c athymic nude mice were injected with 8 × 10^6^ A375 or HPAC cells/100 μL and treated with intratumoral injections of 1 × 10^9^ pfu/50 μL of various types of adenovirus (Ad-NC, Ad-shTGF-β1, or Ad-shTGF-β2) on days 1, 3, and 5. Tumors were collected on day 11 for histological analysis. Paraffin sections of tumor tissue were stained using anti-Hexon, anti-TGF-β1, and anti-TGF-β2 antibodies. **c** A TUNEL assay was performed on tissue sections to quantify apoptotic cell death, as described in the “Materials and methods” section. The percentage of TUNEL-positive cells was determined by counting the TUNEL-positive cells under 10 noncontinuous low-power fields. **d** TGF-β1 or -β2 downregulation can cause both NOX4-mediated ROS production and a reduction in Smad complexes (phospho-Smad2, 3 with Smad4) that translocate to the nucleus to bind to gene promoters for the expression of Trx/GSTM1. ROS triggered by decreased Akt activity can dissociate Trx or GSTM1 from ASK1–Trx and ASK1-GSTM1 complexes. ASK1 activation is also related to the reduction in Trx and GSTM1 gene expression, which results from decreased transcriptional activity of the Smad complex. In these ways, ASK1 was fully activated and induced cytotoxic tumor cell death via p38/JNK activation and induction of ER stress, which stimulated the escalation of ASK1 activation by completion of a sustained positive feedback loop circuit. Circled numbers indicate the chronological order of the signaling events leading to escalated cancer cell death

To examine the in vivo potential of TGF-β shRNA-expressing adenoviruses, we treated nude mice with A375 or HPAC cell grafts. After confirming TGF-β downregulation following treatment with a replication-incompetent adenovirus expressing TGF-β shRNA, replication-incompetent adenoviruses (expressing TGF-β1 or scrambled shRNA as an NC) were tested in nude mice for suppression of tumor growth compared to PBS. Tumor suppression in both cells increased in the order of Ad-shTGF-β1 > Ad-NC > PBS (Fig. 7a, upper). Likewise, the survival rate increased in the order of Ad-shTGF-β1 > Ad-NC > PBS (Fig. 7a, bottom). The expression levels of adenovirus estimated by Hexon staining and TGF-β1 in tumor tissue after infection were as expected, as determined by immunostaining (Fig. 7b). Apoptosis and necrotic events were also increased by an adenovirus containing TGF-β shRNA (Fig. 7c).

In summary, downregulation of TGF-β induces cancer cell death via the ASK1–SAPK axis signaling cascade. Figure 7d provides a schematic diagram summarizing the results.

## Discussion

The expression of TGF-β isoforms is increased in many types of cancers. For example, a high level of TGF-β1 has been detected in gastric cancer^55^, and levels of TGF-β1 and TGF-β2 are markedly increased in hepatocellular carcinoma and pancreatic cancer cells^56,57^. In addition, high TGF-β levels are associated with resistance to anticancer treatments^58^. We found that shRNAs targeting TGF-β1 or TGF-β2 strongly inhibited the growth and survival of tumor cells.

We also found that ASK1 activation was induced by TGF-β downregulation via two separate pathways. One pathway involved decreased gene expression of ASK1-inhibitory binding proteins, and the other functioned through ROS generation and the dissociation of ASK1 protein complexes. Up to the present, Grx and Prx, in addition to Trx and GSTM1, are also known as redox sensors^59–62^. In fact, endogenous cellular Grx levels in various cancer cells, including A375 and HPAC, were rarely detected (data not shown), whereas cellular Prx was detected in higher amounts in A375 cells. However, the cellular Prx level was not decreased, even after TGF-β1 downregulation, and its interaction with ASK1 was rarely detected (Suppl. Figure 5). Therefore, we focused on the explanation of the ASK1–p38/JNK axis after TGF-β downregulation with Trx and GSTM1. Furthermore, we found that TGF-β downregulation repressed the transcription of Trx and GSTM1 via Smad signaling. In fact, in normal cells, Smad-mediated gene responses are not oncogenic, but in cancer cells, they become mediators of malignancy^11^. However, considering that some cancer cells retain functional Smad signaling, the cellular context might determine whether this pathway promotes cancer, as demonstrated by TGF-β signaling^11^.

In the context of physiological/pathophysiological settings, SAPKs are of vital importance to the life or death of a cell^63^. Their effects are determined by the precise nature of the extracellular stimuli and the repertoire of molecules available in the cell, which regulate the localization, timing, intensity, and duration of SAPK activation^64^. Owing to this complexity, the molecular understanding of how JNK and p38 SAPK family members function as either tumor suppressors or oncoproteins in specific cell types remains unknown^65^. There are some reports that JNK and p38 have completely different functions depending on the duration of activation; transient JNK and p38 induction provides a survival signal, whereas persistent activation induces apoptosis^29,66^. In this study, we found that p38 activation (induced by TGF-β downregulation) was particularly related to cancer cell death rather than cell survival due to both the sustained duration and strong intensity of activation. p38 mitogen-activated protein kinase was first known to be activated in response to TGF-β treatment but not to TGF-β downregulation^67–70^. In this case, TGF-β suppressed growth in normal epithelial cells, thereby acting in a cytostatic role to prevent the generation of hyperproliferative disorders such as cancer^1^. However, with an accumulation of genetic and epigenetic alterations in tumor cells, its function switches to the promotion of a pro-invasive and pro-metastatic phenotype, accompanied by a progressive increase in locally secreted TGF-β levels^1^. Owing to the dichotomous nature of TGF-β acting as both a tumor suppressor and a significant stimulator of tumor progression, depending on the cell types in which it is activated, TGF-β treatment or TGF-β downregulation can be used to induce normal cell death or cancer cell death, respectively, of the epithelium through the same signaling pathway. In contrast to TGF-β downregulation in cancer cells, even excessive TGF-β treatment in the same cancer cells did not show any cancer cell death. Instead, TGF-β downregulation was accompanied by weak activation of p38 and a few antiapoptotic molecules, reflecting a low sensitivity of cancer cells with higher levels of TGF-β to TGF-β treatment and again verifying a role of TGF-β as a regulator of cancer cell progression^58,71^. Our results indicating that the differential p38 activation of TGF-β downregulation and TGF-β treatment is correlated with cellular destination are also similar to our previous reports that a biphasic role exists depending on the activation level of p38, that is, cell death with strong p38 activation or cell survival with weak p38 activation after treatment with anticancer agents, such as curcumin or tumor necrosis factor-related apoptosis-inducing ligand^72,73^.

Interestingly, TAK1, which is known to be activated by TGF-β family ligands^23^ and is thought to be a survival signal, did not contribute to the effects observed in this study (data not shown). Rather, we identified ASK1-induced JNK/p-38 as the primary apoptosis signal originating from TGF-β downregulation-induced Akt inactivation. However, it is still difficult to determine whether ASK1–p38/JNK is the only specific key signaling molecule in the SAPK pathway.

Another finding from this study was that the downregulation of TGF-β in cancer cells could promote ROS generation depending on the cancer cell type. TGF-β1 downregulation in A375 cells enhanced ROS in a NOX4-dependent manner, whereas TGF-β2 downregulation in A375 cells or TGF-β1 or -β2 downregulation in HPAC seemed to generate few ROS in a non-NOX4-dependent manner. The increase in ROS in A375 cells after TGF-β1 downregulation was likely triggered by a decrease in tumor-promoting non-canonical Akt activation^25,74^ mediated by NOX4 expression rather than by the canonical Smad signaling pathway, which is also known to have a tumor-promoting role^75^. The cellular redox state is determined by ROS production and elimination under different conditions. (A) Under normal conditions, cancer cells maintain redox homeostasis by balancing ROS production and elimination. (**B**) Under metabolic stress, redox homeostasis is damaged owing to enhanced ROS production and decreased ROS elimination^76^. Based on these two possible environments, lower levels of ROS can oxidize the disulfide bridges in Akt, leading to the association of Akt with PP2A and thus short-term activation of Akt^77^. However, because higher levels of ROS correspond to an overall decrease in cell survival potential, including Akt inactivation, resulting in severe damage to survival–death homeostasis, ROS are likely to be an irreversible sign of cell death, exacerbating ASK1-induced cell death through enhanced ER stress conditions.

Taken together, our findings demonstrated that the downregulation of TGF-β via shRNAs induced tumor cell death, an effect that was driven by ASK1–SAPK axis signaling cascade regulated by a positive feedback circuit triggered by Akt inactivation/NOX4 increase-derived ROS-mediated ER stress (A375 cells) or tumor cell death driven by direct ASK1–SAPK axis signaling cascade (HPAC cells). For the goal of cancer treatment, it would be desirable to retain the TGF-β-mediated stimulation of apoptosis in tumor cells but inhibit the cell-autonomous and non-cell-autonomous activities of TGF-β^8^. In light of this viewpoint, downregulation of TGF-β rather than knockout might result in a more effective therapeutic regimen. However, it is challenging to predict whether the blockade of TGF-β with inhibitors would be sufficient to block the tumor-promoting effects of TGF-β without affecting its tumor-suppressive functions^78^. Nevertheless, our data provide an underlying mechanism of how TGF-β downregulation induces cancer cell death.

## Electronic supplementary material

Supplementary legends

Supplementary figure 1

Supplementary figure 2

Supplementary figure 3

Supplementary figure 4

Supplementary figure 5
