# Supplementary material for: TGF-β downregulation-induced cancer cell death is finely regulated by the SAPK signaling cascade
Source: Exp Mol Med. 2018 Dec 6;50(12):162. doi: 10.1038/s12276-018-0189-8 (PMC6283885; doi:10.1038/s12276-018-0189-8)
Supplement: Supplementary file 1 — Supplementary legends [file 12276_2018_189_MOESM1_ESM.docx]

**Supplementary figure 1. Screening of human TGF-β1 and TGF-β2 shRNAs. A.** Sequences of shRNA oligomers targeting human TGF-β1 are shown with the selected target sequence indicated in bold (Top). The candidate shRNA oligomers for each target and the negative or positive control shRNA were transfected into HeLa cells. The knockdown efficiency of each oligomer was measured using quantitative real-time PCR to amplify human TGF-β1. Relative expression levels of human TGF-β1 were plotted after normalization to the scrambled shRNA as a negative control (Bottom). **B.** Sequences of shRNA oligomers targeting human TGF-β2 are shown with the selected target sequence indicated in bold (Top). The candidate shRNA oligomers for each target and the negative or positive control shRNA were transfected into HeLa cells. The knockdown efficiency of each oligomer was measured using quantitative real-time PCR to amplify human TGF-β2. Relative expression levels of human TGF-β2 were plotted after normalization to the scrambled shRNA as a negative control (Bottom).

**Supplementary figure 2. Transcriptome profling analysis in two different cancer cells by scatter plots of probe set intensities.** Normalized probe set intensities of two cancer cell lines are plotted after 48 h infection of TGF-β1 or β2 shRNA-expressing adenovirus. The numbers indicate over 1.5 fold variation in intensities outside of two diagonal lines among 21,448 probe sets.

**Supplementary figure 3. No off-targeting effect of adenovirus-expressing shTGF-β1 in melanoma cancer cell lines.** **A.** A375 cell lines were infected with adenovirus-expressing shTGF-β1 at 100 MOI with or without of recombinant TGF-β1 750 ng/ml for time-dependent, respectively. TGF-β1 (0 h) means TGF-β1 protein was treated with adenovirus-expressing shTGF-β1, simultaneously. After 48 h, the expressions of p-p38, p-Akt, p-HSP27, p-ERK, p-src, p-p65, p-stat3 and GAPDH were detected by western blot analysis. **B.** A375 cell lines were infected with adenovirus-expressing shTGF-β1 at 100 MOI with or without of recombinant TGF-β1 750 ng/ml for time-dependent, respectively. TGF-β1 (0 h) means TGF-β1 protein was treated with adenovirus-expressing shTGF-β1, simultaneously. After 48 h, morphological changes were observed by using microscopy. **C.** A375 cell lines were infected with adenovirus-expressing shTGF-β2 at 100 MOI with or without of recombinant TGF-β2 750 ng/ml for time-dependent, respectively. TGF-β2 (0 h) means TGF-β2 protein was treated with adenovirus-expressing shTGF-β2, simultaneously. After 48 h, the expressions of p-p38, p-Akt, p-HSP27, p-ERK, p-src, p-p65, p-stat3 and GAPDH were detected by western blot analysis. **D.** A375 cell lines were infected with adenovirus-expressing shTGF-β2 at 100 MOI with or without of recombinant TGF-β2 750 ng/ml for time-dependent, respectively. TGF-β2 (0 h) means TGF-β2 protein was treated with adenovirus-expressing shTGF-β2, simultaneously. After 48 h, morphological changes were observed by using microscopy.

**Supplementary figure 4. Effect of TGF-β1 treatment on A375 and HPAC cancer cells. A.** A375 and HPAC cells were treated with TGF-β1 protein in dose-dependent manner (5 ng/ml ~ 500 ng/ml) for 1 h and the expressions of p-p38, p38, p-Akt, p-src, p-p65, p-stat3 and GAPDH were detected via western blot analysis. **B**. A375 and HPAC cells were treated with TGF-β1 protein (100 ng/ml, 500 ng/ml) for 24 h or 48 h and morphological changes were observed using microscopy.

**Supplementary figure 5. Prx expression after TGF-β downregulation and interaction with ASK1. A*.*** A375 cells were infected with adenovirus-expressing shTGF-β1 or 2 at 100 MOI, respectively. After 48 h, the expressions of phospho-p38, p38, Prx, and GAPDH were detected by western blot analysis. **B.** Lysates of A375 cells were subjected to immunoprecipitation with using an anti‐Trx (Left) or Prx (Right) antibody to identify interaction with ASK1. Interaction between Trx and ASK1 was used as a positive control.
