# Supplementary material for: TGF-β downregulation-induced cancer cell death is finely regulated by the SAPK signaling cascade
Source: Exp Mol Med. 2018 Dec 6;50(12):162. doi: 10.1038/s12276-018-0189-8 (PMC6283885; doi:10.1038/s12276-018-0189-8)
Supplement: Supplementary file 2 — Supplementary figure 1 [file 12276_2018_189_MOESM2_ESM.pptx]

## Slide 1
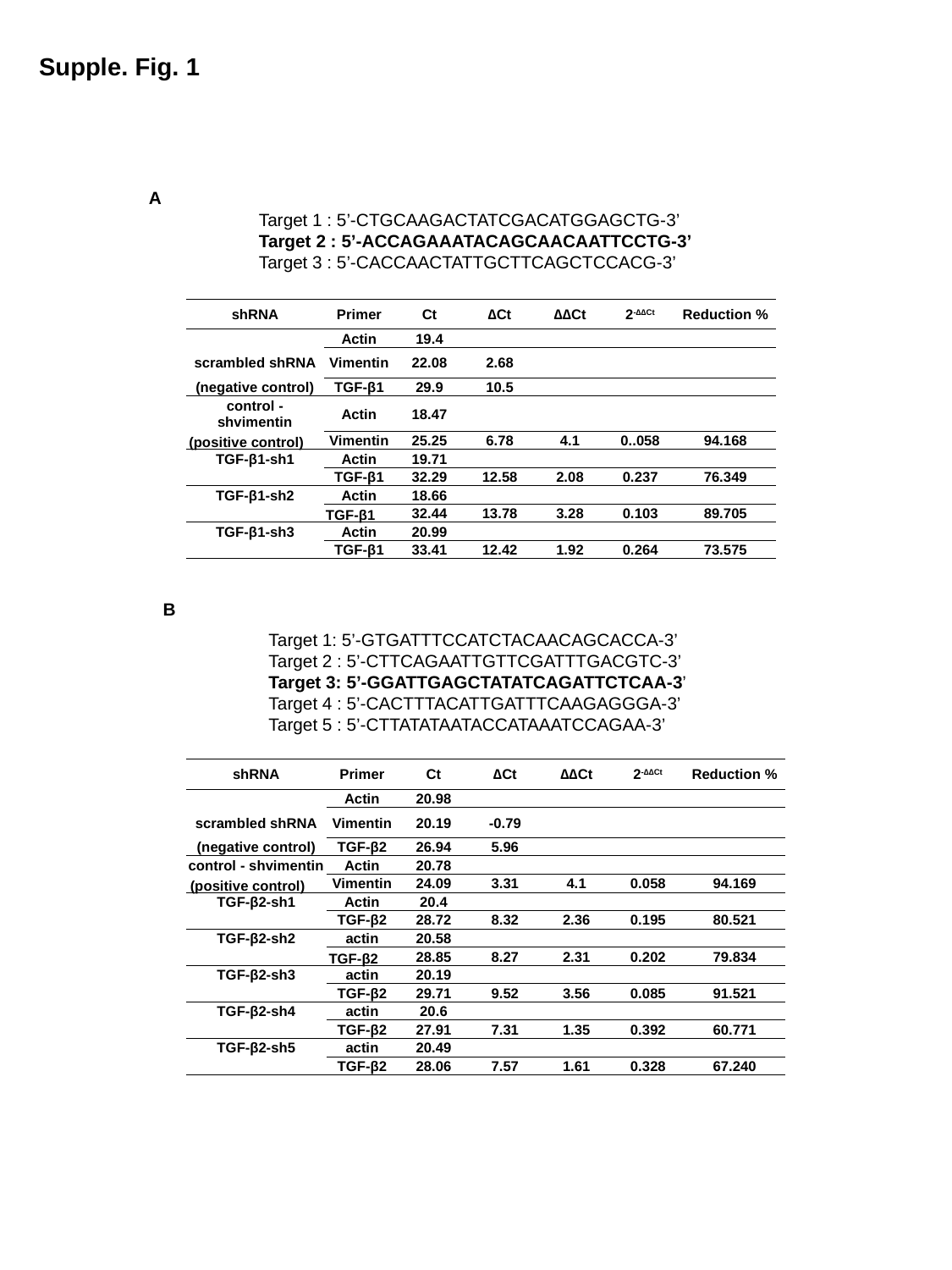

Supple. Fig. 1
A
Target 1 : 5’-CTGCAAGACTATCGACATGGAGCTG-3’
Target 2 : 5’-ACCAGAAATACAGCAACAATTCCTG-3’
Target 3 : 5’-CACCAACTATTGCTTCAGCTCCACG-3’
| shRNA | Primer | Ct | ∆Ct | ∆∆Ct | 2-∆∆Ct | Reduction % |
| --- | --- | --- | --- | --- | --- | --- |
| | Actin | 19.4 | | | | |
| scrambled shRNA | Vimentin | 22.08 | 2.68 | | | |
| (negative control) | TGF-β1 | 29.9 | 10.5 | | | |
| control - shvimentin | Actin | 18.47 | | | | |
| (positive control) | Vimentin | 25.25 | 6.78 | 4.1 | 0..058 | 94.168 |
| TGF-β1-sh1 | Actin | 19.71 | | | | |
| | TGF-β1 | 32.29 | 12.58 | 2.08 | 0.237 | 76.349 |
| TGF-β1-sh2 | Actin | 18.66 | | | | |
| | TGF-β1 | 32.44 | 13.78 | 3.28 | 0.103 | 89.705 |
| TGF-β1-sh3 | Actin | 20.99 | | | | |
| | TGF-β1 | 33.41 | 12.42 | 1.92 | 0.264 | 73.575 |
B
Target 1: 5’-GTGATTTCCATCTACAACAGCACCA-3’
Target 2 : 5’-CTTCAGAATTGTTCGATTTGACGTC-3’
Target 3: 5’-GGATTGAGCTATATCAGATTCTCAA-3’
Target 4 : 5’-CACTTTACATTGATTTCAAGAGGGA-3’
Target 5 : 5’-CTTATATAATACCATAAATCCAGAA-3’
| shRNA | Primer | Ct | ∆Ct | ∆∆Ct | 2-∆∆Ct | Reduction % |
| --- | --- | --- | --- | --- | --- | --- |
| | Actin | 20.98 | | | | |
| scrambled shRNA | Vimentin | 20.19 | -0.79 | | | |
| (negative control) | TGF-β2 | 26.94 | 5.96 | | | |
| control - shvimentin | Actin | 20.78 | | | | |
| (positive control) | Vimentin | 24.09 | 3.31 | 4.1 | 0.058 | 94.169 |
| TGF-β2-sh1 | Actin | 20.4 | | | | |
| | TGF-β2 | 28.72 | 8.32 | 2.36 | 0.195 | 80.521 |
| TGF-β2-sh2 | actin | 20.58 | | | | |
| | TGF-β2 | 28.85 | 8.27 | 2.31 | 0.202 | 79.834 |
| TGF-β2-sh3 | actin | 20.19 | | | | |
| | TGF-β2 | 29.71 | 9.52 | 3.56 | 0.085 | 91.521 |
| TGF-β2-sh4 | actin | 20.6 | | | | |
| | TGF-β2 | 27.91 | 7.31 | 1.35 | 0.392 | 60.771 |
| TGF-β2-sh5 | actin | 20.49 | | | | |
| | TGF-β2 | 28.06 | 7.57 | 1.61 | 0.328 | 67.240 |
