# Supplementary material for: TGF-β downregulation-induced cancer cell death is finely regulated by the SAPK signaling cascade
Source: Exp Mol Med. 2018 Dec 6;50(12):162. doi: 10.1038/s12276-018-0189-8 (PMC6283885; doi:10.1038/s12276-018-0189-8)
Supplement: Supplementary file 3 — Supplementary figure 2 [file 12276_2018_189_MOESM3_ESM.pptx]

## Slide 1
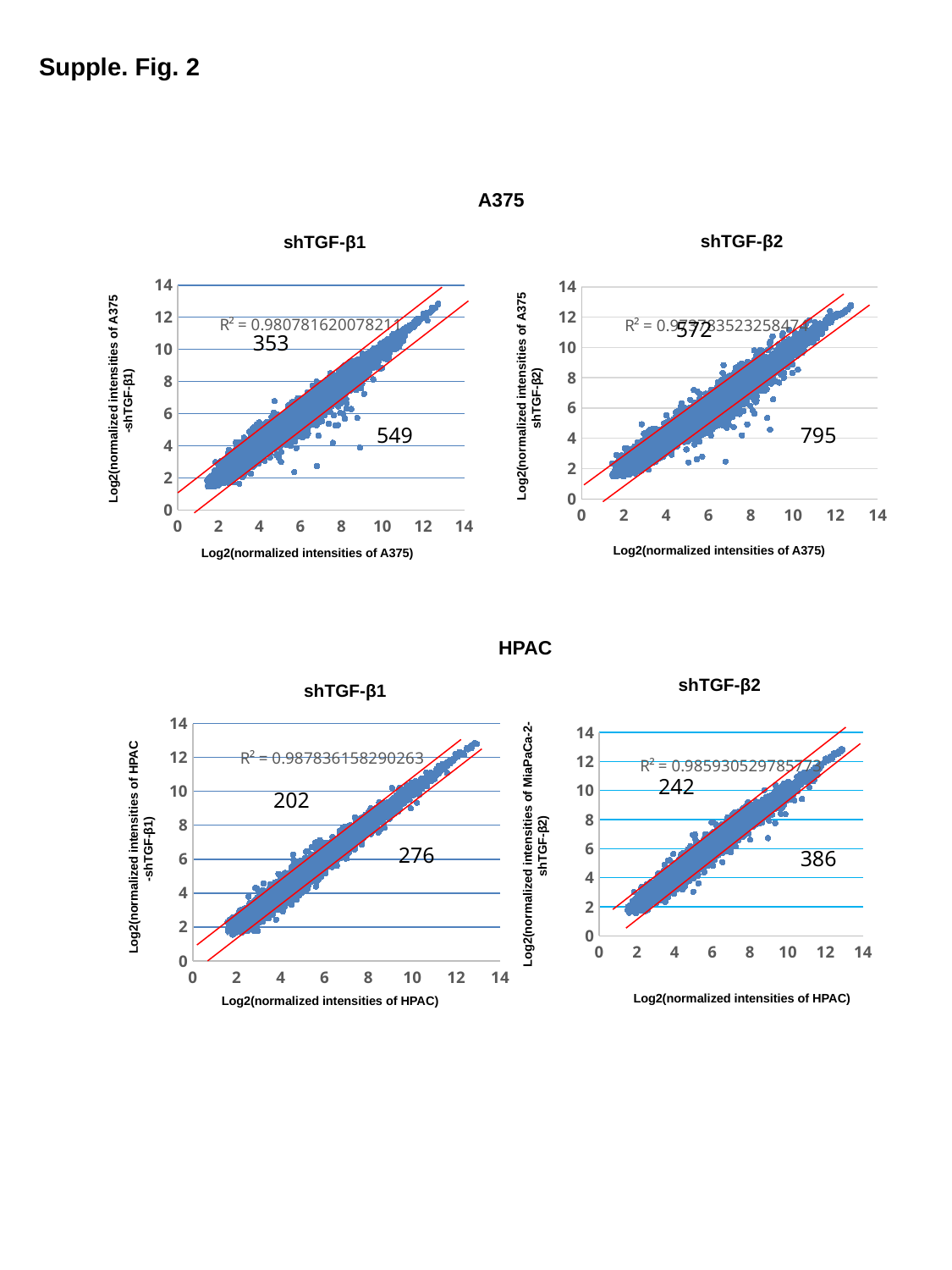

Supple. Fig. 2
A375
shTGF-β2
### Chart
| Category | N_b1 |
|---|---|shTGF-β1
### Chart
| Category | N_b2 |
|---|---|572
353
Log2(normalized intensities of A375
shTGF-β2)
Log2(normalized intensities of A375
-shTGF-β1)
549
795
Log2(normalized intensities of A375)
Log2(normalized intensities of A375)
HPAC
shTGF-β2
shTGF-β1
### Chart
| Category | N_HP_NC |
|---|---|
### Chart
| Category | N_HP_NC |
|---|---|242
202
Log2(normalized intensities of MiaPaCa-2-
shTGF-β2)
Log2(normalized intensities of HPAC
-shTGF-β1)
276
386
Log2(normalized intensities of HPAC)
Log2(normalized intensities of HPAC)
