# Supplementary material for: TGF-β downregulation-induced cancer cell death is finely regulated by the SAPK signaling cascade
Source: Exp Mol Med. 2018 Dec 6;50(12):162. doi: 10.1038/s12276-018-0189-8 (PMC6283885; doi:10.1038/s12276-018-0189-8)
Supplement: Supplementary file 4 — Supplementary figure 3 [file 12276_2018_189_MOESM4_ESM.pptx]

## Slide 1
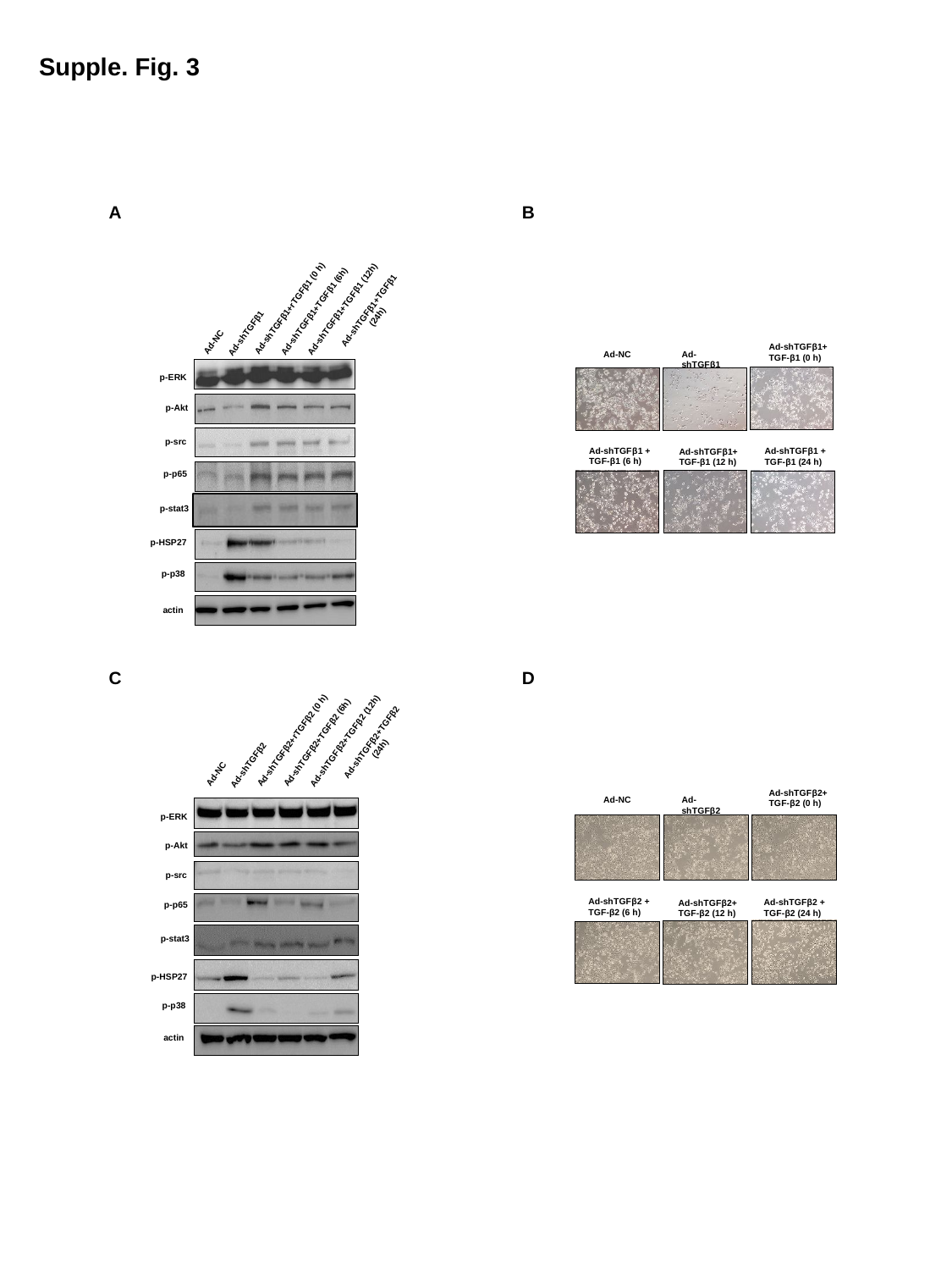

Supple. Fig. 3
A
B
Ad-shTGFβ1+rTGFβ1 (0 h)
Ad-shTGFβ1+TGFβ1 (12h)
Ad-shTGFβ1+TGFβ1 (24h)
Ad-shTGFβ1+TGFβ1 (6h)
Ad-shTGFβ1
Ad-NC
Ad-shTGFβ1+
TGF-β1 (0 h)
Ad-NC
Ad-shTGFβ1
p-ERK
p-Akt
p-src
Ad-shTGFβ1 +
TGF-β1 (6 h)
Ad-shTGFβ1 +
TGF-β1 (24 h)
Ad-shTGFβ1+
TGF-β1 (12 h)
p-p65
p-stat3
p-HSP27
p-p38
actin
C
D
Ad-shTGFβ2+rTGFβ2 (0 h)
Ad-shTGFβ2+TGFβ2 (12h)
Ad-shTGFβ2+TGFβ2 (24h)
Ad-shTGFβ2+TGFβ2 (6h)
Ad-shTGFβ2
Ad-NC
Ad-shTGFβ2+
TGF-β2 (0 h)
Ad-NC
Ad-shTGFβ2
p-ERK
p-Akt
p-src
Ad-shTGFβ2 +
TGF-β2 (6 h)
Ad-shTGFβ2 +
TGF-β2 (24 h)
Ad-shTGFβ2+
TGF-β2 (12 h)
p-p65
p-stat3
p-HSP27
p-p38
actin
