# Supplementary material for: TGF-β downregulation-induced cancer cell death is finely regulated by the SAPK signaling cascade
Source: Exp Mol Med. 2018 Dec 6;50(12):162. doi: 10.1038/s12276-018-0189-8 (PMC6283885; doi:10.1038/s12276-018-0189-8)
Supplement: Supplementary file 5 — Supplementary figure 4 [file 12276_2018_189_MOESM5_ESM.pptx]

## Slide 1
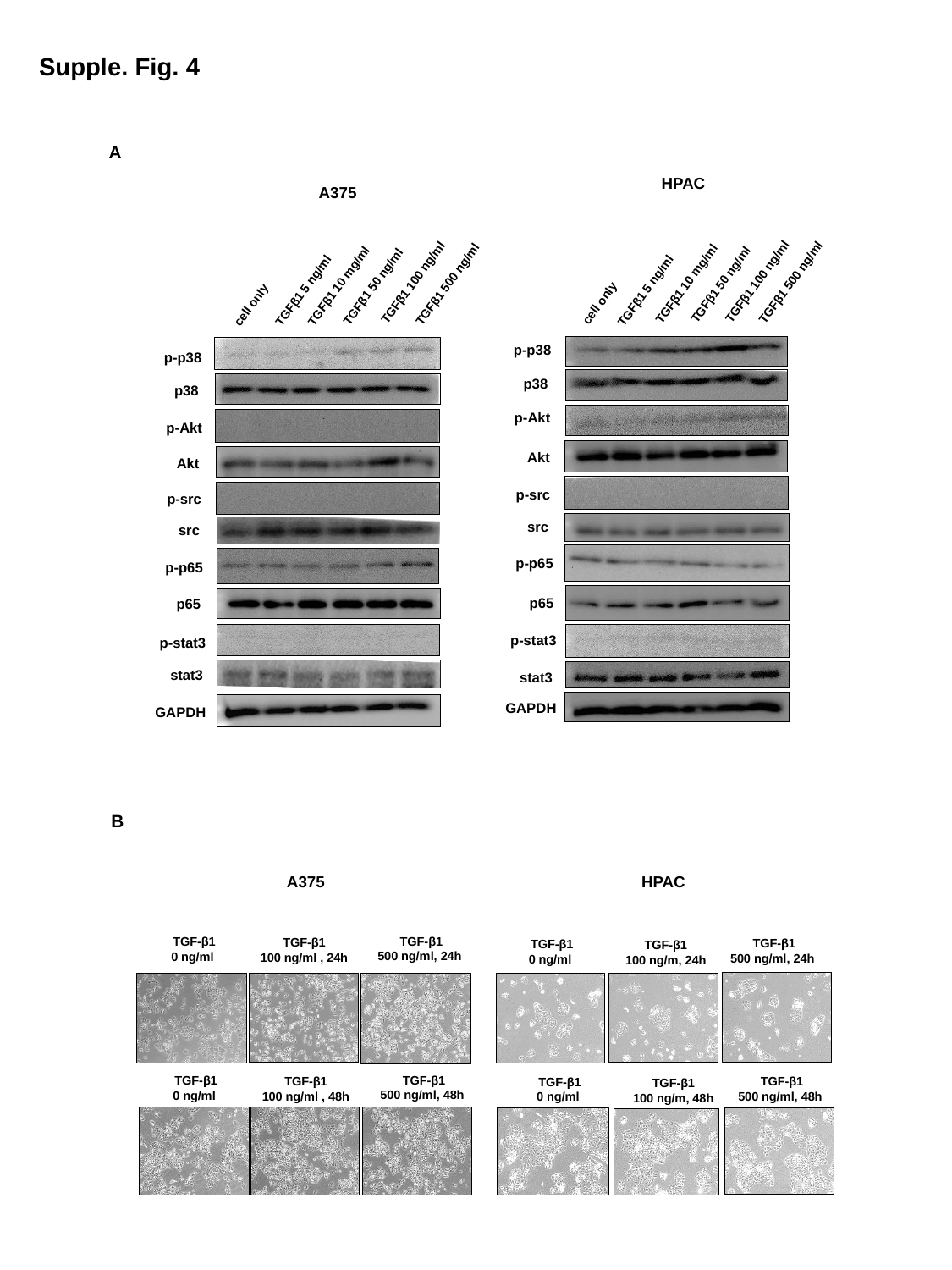

Supple. Fig. 4
A
HPAC
A375
TGFβ1 100 ng/ml
TGFβ1 500 ng/ml
TGFβ1 100 ng/ml
TGFβ1 10 mg/ml
TGFβ1 500 ng/ml
TGFβ1 50 ng/ml
TGFβ1 10 mg/ml
TGFβ1 50 ng/ml
TGFβ1 5 ng/ml
TGFβ1 5 ng/ml
cell only
cell only
p-p38
p-p38
p38
p38
p-Akt
p-Akt
Akt
Akt
p-src
p-src
src
src
p-p65
p-p65
p65
p65
p-stat3
p-stat3
stat3
stat3
GAPDH
GAPDH
X10.0
B
A375
HPAC
TGF-β1
500 ng/ml, 24h
TGF-β1
0 ng/ml
TGF-β1
100 ng/ml , 24h
TGF-β1
500 ng/ml, 24h
TGF-β1
0 ng/ml
TGF-β1
100 ng/m, 24h
TGF-β1
500 ng/ml, 48h
TGF-β1
0 ng/ml
TGF-β1
500 ng/ml, 48h
TGF-β1
100 ng/ml , 48h
TGF-β1
0 ng/ml
TGF-β1
100 ng/m, 48h
