# Supplementary material for: TGF-β downregulation-induced cancer cell death is finely regulated by the SAPK signaling cascade
Source: Exp Mol Med. 2018 Dec 6;50(12):162. doi: 10.1038/s12276-018-0189-8 (PMC6283885; doi:10.1038/s12276-018-0189-8)
Supplement: Supplementary file 6 — Supplementary figure 5 [file 12276_2018_189_MOESM6_ESM.pptx]

## Slide 1
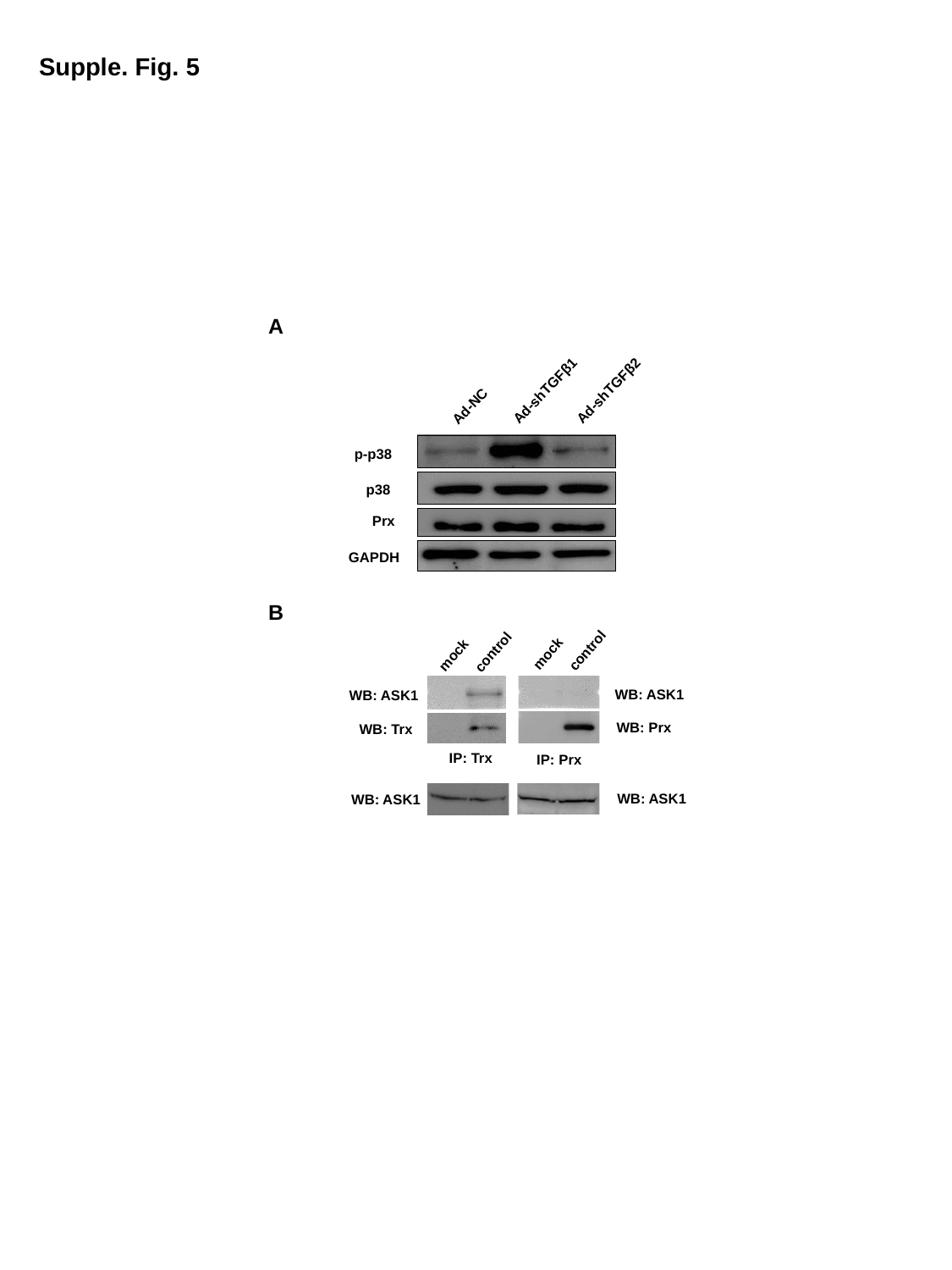

Supple. Fig. 5
A
Ad-shTGFβ1
Ad-shTGFβ2
Ad-NC
p-p38
p38
Prx
GAPDH
B
control
control
mock
mock
WB: ASK1
WB: ASK1
WB: Prx
WB: Trx
IP: Trx
IP: Prx
WB: ASK1
WB: ASK1
